# Exosomal lncRNA FENDRR Orchestrates Immune Remodelling and Ferroptosis in the Comorbidity of Lung Cancer and Type 1 Myocardial Infarction

**DOI:** 10.1155/humu/8861747

**Published:** 2026-01-23

**Authors:** Youfu He, Yu Qian, Zhiwei Zheng, Yu Zhou, Chen Li, Qiang Wu

**Affiliations:** ^1^ Department of Cardiology, Guizhou Provincial People′s Hospital, Guiyang City, Guizhou Province, China, 5055.cn; ^2^ Medical College, Guizhou University, Guiyang City, Guizhou Province, China, gzu.edu.cn; ^3^ Department of Cardiology, Guizhou Provincial Key Laboratory of Pathogenesis and Prevention of Common Chronic Diseases, Guiyang City, Guizhou Province, China; ^4^ Department of Cardiology, The Second Affiliated Hospital of Zunyi Medical University, Zunyi City, Guizhou Province, China, zmc.edu.cn; ^5^ Department of Pharmacy, The First Affiliated Hospital of Guangxi Medical University, Nanning City, Guangxi Province, China, gxmu.edu.cn

**Keywords:** exosomes, FENDRR, immune microenvironment, lung cancer, Type 1 myocardial infarction

## Abstract

Lung cancer and Type 1 myocardial infarction (T1MI) increasingly co‐occur, yet the molecular basis underlying their interaction remains unclear. In this study, we combined multiomics profiling, in vivo and in vitro models and human clinical samples to investigate the regulatory role of the exosomal long noncoding RNA FENDRR in cancer–cardiovascular comorbidity. We found that FENDRR was markedly elevated in thrombus‐derived exosomes from T1MI patients and promoted cardiomyocyte ferroptosis and ferritinophagy through the NCOA4–GPX4–P62 axis, thereby exacerbating myocardial injury. Silencing FENDRR significantly alleviated cardiac damage in the T1MI rat model. In contrast, FENDRR was consistently downregulated across multiple cancers, particularly lung adenocarcinoma (LUAD). Higher FENDRR expression was associated with favourable patient survival, and time‐dependent ROC analysis demonstrated robust prognostic performance in LUAD (5 − year AUC = 0.990). Multiomics and immunogenomic analyses further revealed that FENDRR expression correlated with distinct remodelling of the tumour immune microenvironment, including alterations in immune cell infiltration, immune activation scores, chemokine and HLA gene expression and antigen‐presentation capacity. These findings were supported by single‐cell analyses and by enhanced CD8^+^ T‐cell and Treg infiltration in thrombi from patients with LUAD and T1MI. Collectively, our results identify FENDRR as a context‐dependent regulator that promotes myocardial injury but may exert tumour‐suppressive and immune‐modulatory functions in lung cancer. These insights provide a mechanistic framework for cancer–cardiovascular comorbidity and highlight FENDRR as a potential biomarker and therapeutic target across disease contexts.

## 1. Introduction

Acute myocardial infarction (AMI), particularly Type 1 myocardial infarction (T1MI), has long remained the leading cause of cardiovascular mortality worldwide owing to its high incidence and lethality [1]. T1MI primarily results from the rupture of coronary atherosclerotic plaques and subsequent thrombus formation, and it is most commonly observed among elderly individuals and those with multiple chronic diseases [2]. In recent years, with advances in cancer therapy and an ageing population, the phenomenon of comorbidity between cardiovascular disease and malignancy has become increasingly prominent [3–9], thereby attracting considerable attention in both basic and clinical research.

Lung cancer, as one of the leading causes of cancer‐related death worldwide, continues to exhibit a rising incidence [10]. Clinical epidemiological data indicate that patients with lung cancer are at a significantly higher risk of developing AMI, particularly T1MI, compared with the general population, and AMI has emerged as one of the most critical complications impacting the prognosis of lung cancer patients [11]. The coexistence of these two conditions not only exacerbates the overall disease burden, but also significantly compromises survival and quality of life [12, 13]. The underlying interactive mechanisms are highly complex, involving not only traditional risk factors such as smoking, chronic inflammation and metabolic abnormalities, but also cancer‐related metabolic disorders, treatment‐associated toxicity and haematological abnormalities [14].

However, the molecular mechanisms and signalling networks behind the comorbidity of lung cancer and T1MI remain unclear. Against this background, exosomes and their long noncoding RNA (lncRNA) cargo have become a major focus in studies of intercellular communication [15]. As extracellular vesicles released by cells, exosomes can transport a variety of active molecules including proteins, miRNAs and lncRNAs throughout the circulatory system, thereby enabling long‐range intercellular communication [16]. Extensive studies have demonstrated that exosomes derived from tumours and thrombi play irreplaceable roles in regulating vascular function, immune responses, apoptosis and interorgan injury ([7]). In the context of cancer–cardiovascular comorbidity, exosomal lncRNAs have been shown to promote cardiovascular injury and tumour progression by remodelling the immune microenvironment, mediating signal transduction or modulating cellular stress responses [17, 18].

Of particular interest, both immune‐related mechanisms and those associated with ferroptosis and ferritinophagy play central roles in the development and progression of malignancy and myocardial infarction. Immune cell infiltration, cytokine secretion and immune checkpoint expression not only influence thrombosis and myocardial injury but also profoundly shape the initiation, progression and immune evasion of cancer. Meanwhile, ferroptosis and ferritinophagy, two recently described processes involved in regulated cell death and iron handling, have been linked to both myocardial injury and tumour progression. Molecules associated with ferroptosis, such as GPX4, NCOA4 and P62, can modulate oxidative stress and immune signalling, exacerbating tissue injury or influencing the local microenvironment [18]. The interactive regulation among immunity, ferroptosis and exosomal lncRNAs provides a novel theoretical framework for understanding cancer–myocardial infarction comorbidity, though the detailed molecular mechanisms have yet to be systematically elucidated.

In this study, we combined multiomics analyses, in vivo and in vitro models, and clinical specimens to examine thrombus‐derived exosomal lncRNAs in patients with lung cancer and concomitant T1MI, with a particular focus on fetal–lethal noncoding developmental regulatory RNA (FENDRR). We then explored the downstream regulatory networks of FENDRR and its effects on the immune microenvironment and on ferroptosis and ferritinophagy pathways. The contribution of FENDRR to myocardial injury and to the link between cancer and cardiovascular disease was further supported by animal and cell‐based experiments. Together, these findings outline potential molecular targets and provide mechanistic insight that may help refine risk stratification and guide future diagnostic and therapeutic strategies in cancer–cardiovascular comorbidity.

## 2. Materials and Methods

### 2.1. Isolation and Characterisation of Thrombus‐Derived Exosomes (TEs)

TEs were isolated as previously described ([4–6]). Briefly, fresh thrombus tissue was homogenised in PBS, followed by a low‐speed centrifugation to remove cellular debris. The supernatant was then centrifuged at 10,000 × g for 30 min, collected and subjected to ultracentrifugation at 100,000 × g for 70 min. The resulting pellet was resuspended in PBS, filtered through a 0.22‐*μ*m membrane, and ultracentrifuged again. Exosome identity was confirmed by three methods:

1. Western blotting: detection of exosome markers CD63 (Cat# 25682‐1‐AP) and CD9 (Cat# 20597‐1‐AP) using antibodies from Wuhan Sanying;
2. Nanoparticle tracking analysis (NTA): assessment of size distribution and concentration (ZetaView PMX120, Particle Metrix); and
3. Transmission electron microscopy (TEM): visualisation of typical cup‐shaped vesicle structures.

### 2.2. lncRNA Microarray and Expression Profiling

Exosomal RNA was extracted using the TRIzol method (Invitrogen, United States), and quantified and assessed by Sangon Biotech (Shanghai, China). The microarray platform was an Agilent human lncRNA microarray (details in supporting information), and expression data were normalised using the RMA method. Differential expression was analysed with the limma package (v3.54.2). DElncRNAs were defined as those with |log2FC| > 1 and adjusted *p* < 0.05. Selected lncRNAs were validated by qRT‐PCR using primers synthesised by Sangon.

### 2.3. Functional Enrichment and Pathway Analysis of Differentially Expressed Genes

DElncRNA target gene enrichment was performed using the R package clusterProfiler (v4.6.2), including GO (BP/CC/MF), KEGG and GSEA analyses. GSEA used the MSigDB v2023.1 gene sets, with FDR < 0.25 and *p* < 0.05 indicating significance. Results were visualised using barplots, enrichment maps and heat maps [19, 20].

### 2.4. Cell Culture and Functional Assays

The human cardiomyocyte line AC16 was cultured in DMEM supplemented with 10% FBS (Gibco, United States) at 37°C and 5% CO_2_. Experimental groups included control, TE treatment, TE + si − FENDRR and TE + OE − FENDRR. FENDRR siRNA was synthesized by Hanheng Biotechnology (sense: GGCUGAUGGUAGAGGUUAAAC; antisense: UUAACCUCUACCAUCAGCCGG), and the shRNA targeting FENDRR had the following sequences: top strand: 5 ^′^‐CACCGGAGGAAGAGAAGTATCAATTCGAAAATTGATACTTCTCTTCCTCC‐3 ^′^; bottom strand: 5 ^′^‐AAAAGGAGGAAGAGAAGTATCAATTTTCGAATTGATACTTCTCTTCCTCC‐3 ^′^, designed using Block‐iT RNAi Designer. The OE‐FENDRR vector was constructed in pcDNA3.1‐FENDRR and synthesized by Hanheng Biotechnology, with sequencing confirmation.

Transfections were carried out with Lipofectamine 3000 (Invitrogen) according to the manufacturer′s protocol. Cell viability was measured with the CCK8 assay (Beyotime, Cat# C0037); apoptosis was assessed using the Annexin V‐FITC/PI apoptosis detection kit (Beyotime, Cat# C1062L), and ROS levels were measured using the DCFH‐DA probe (Beyotime, Cat# S0033S). Ferroptosis and ferritinophagy proteins (GPX4, NCOA4 and P62) were detected by Western blot using primary antibodies from Wuhan Sanying (GPX4: Cat# 67763‐1‐Ig; NCOA4: Cat# 83394‐4‐RR; P62: Cat# 18420‐1‐AP) and HRP‐conjugated goat anti‐rabbit IgG secondary antibody (Wuhan Sanying, Cat# SA00001‐2).

### 2.5. Establishment and Histological Evaluation of the T1MI Animal Model

T1MI was induced in healthy male Sprague–Dawley rats (8 weeks old, 200–250 g) under anaesthesia and aseptic conditions. After thoracotomy and cardiac exposure, a filter paper (2 × 5 mm) soaked in 5% FeCl_3_ was gently applied to the left anterior descending (LAD) artery for 5 min, then removed, followed by haemostasis and wound closure. ST‐segment elevation within 10–20 s on ECG was considered indicative of successful modelling [21]. All animal protocols were approved by the institutional ethics committee.

Rats were divided into sham, T1MI and shFENDRR intervention groups and sacrificed 24–48 h after surgery. Cardiac tissues were stained with haematoxylin and eosin (HE; Beyotime, Cat# G1120) and Masson′s trichrome (Beyotime, Cat# G1340) for assessment of pathological injury and fibrosis. Ferroptosis markers MDA and SOD were measured using kits from Nanjing Jiancheng (MDA: Cat# A003‐1‐2; SOD: Cat# A001‐3‐2), and Fe^2+^ was measured with a Beyotime kit (Cat# S1066S).

### 2.6. RT‐qPCR and Western Blot Analysis

Total RNA was extracted with TRIzol (Invitrogen, United States), reverse transcribed with the Takara kit (Takara, Cat# RR036A) and amplified using SYBR Green qPCR Master Mix (Takara, Cat# RR820A). All gene primers were synthesized by Sangon (sequences in supporting information); *β*‐actin was used as the internal control. Western blots were performed by standard SDS‐PAGE, with antibodies as described above.

### 2.7. Oncological Omics and Machine Learning Analysis

Tumour datasets from TCGA and GEO were standardised and analysed for differential expression using DESeq2 and limma. Survival analyses and prognostic modelling were performed with COX regression, Lasso, CoxBoost and Ridge algorithms [4–6], in R 4.2.2 and Python 3.10. AUC and C‐index curves were plotted using pROC (v1.18.4) and survival (v3.5‐5) packages. Protein interaction networks were analysed with STRING (v12.0) and Cytoscape (v3.9.1).

### 2.8. Immune Microenvironment and Single‐Cell Analysis

Immune infiltration was evaluated using CIBERSORT (v1.04), TIMER (v2.0), EPIC and ssGSEA. TIP, Tcell‐inflamed, TLS and MeTIL scores were calculated based on published signatures. Single‐cell transcriptome analysis was conducted using the standard Seurat workflow (v4.3.0) with UMAP for dimensionality reduction, clustering and lncRNA/marker gene visualization. All R packages and code are publicly available on GitHub.

### 2.9. Immunohistochemistry and Quantitative Analysis

Coronary thrombus specimens were paraffin‐embedded and sectioned at 4 *μ*m. IHC was performed to detect CD8 (Wuhan Sanying, Cat# 66868‐1‐Ig), CXCL8 (Cat# 27095‐1‐AP), PTX3 (Cat# 13797‐1‐AP), PD‐L1 (Cat# 28076‐1‐AP) and FOXP3 (Cat# 22228‐1‐AP), with DAB chromogen (Beyotime, Cat# P0202). Images were captured using a Leica DM2500 microscope and analyzed with ImageJ (v1.54) and the IHC Toolbox plugin for DAB signal separation, using unified thresholds. Integrated density and positive area ratio were calculated; the mean values were used for statistical analysis.

### 2.10. Ethics Statement

All clinical specimens and animal experiments were approved by the institutional ethics committee. Human and animal studies conformed to the Declaration of Helsinki and Chinese animal welfare regulations. Informed consent was obtained from all participants.

### 2.11. Statistical Analysis

All data are presented as mean ± standard deviation (mean ± SD). Student′s *t*‐test was used for two‐group comparisons; nonparametric data were analysed with the Mann–Whitney *U* test. Comparisons among multiple groups were made using one‐way ANOVA. Survival curves were compared using the log–rank test; COX regression was employed for prognostic factor screening. All statistical analyses were performed with R 4.2.2 and GraphPad Prism 9.0 (GraphPad Software, United States). Two‐sided *p* < 0.05 was considered significant.

## 3. Results

### 3.1. Isolation and Characterisation of TEs

Western blotting for exosomal surface markers CD63 and CD9 confirmed the classical protein expression profile of the isolated TEs (Figure 1a). NTA demonstrated that the diameter of TEs was mainly distributed between 50 and 200 nm (Figure 1b), with the vast majority centred at approximately 125.1 nm and a FWHM of 98.5%, indicating a highly homogeneous exosome population (Figure 1c). TEM revealed that TEs possessed typical cup‐shaped or rounded vesicular structures, with diameters of around 100 nm (Figure 1d).

Figure 1Extraction and characterisation of thrombus‐derived exosomes (TEs). (a) Western blot analysis validated the expression of exosomal surface markers. (b) NTA determined the particle size range of TEs. (c) Peak analysis by NTA. (d) Electron microscopy identified the typical morphology of TEs (× 85,000).

**Figure:**
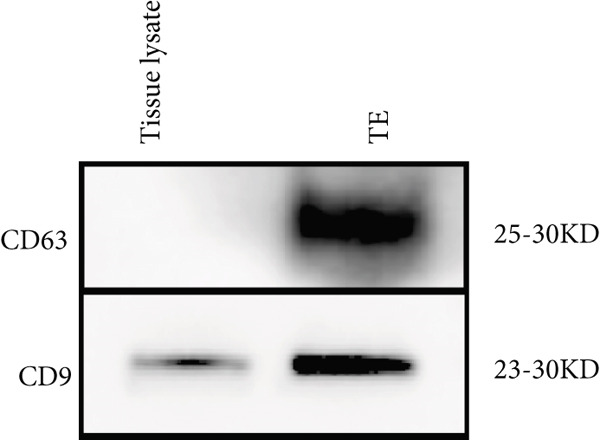
(a)

**Figure:**
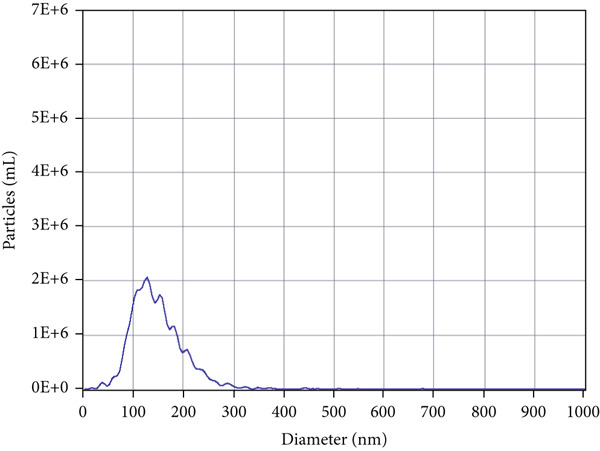
(b)

**Figure:**
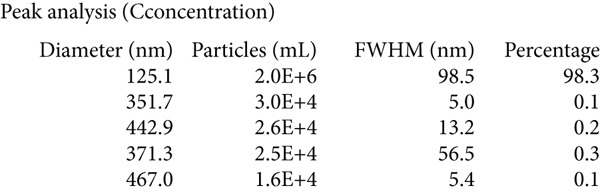
(c)

**Figure:**
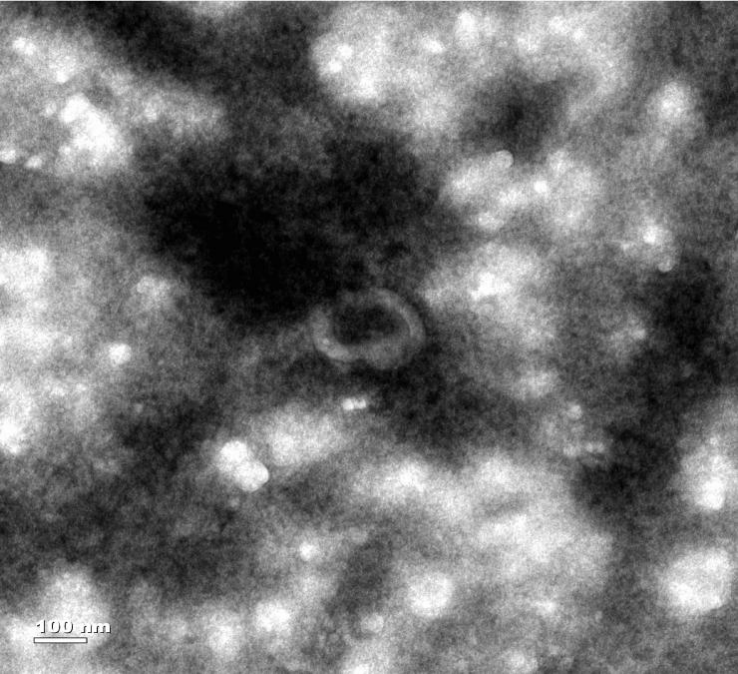
(d)

### 3.2. Exosomal lncRNA Profiling and Functional Enrichment

Microarray analysis revealed a large number of differentially expressed lncRNAs between TEs from myocardial infarction patients and serum exosomes from healthy individuals (normal) (Figure 2a). GSEA demonstrated significant differences in functional distribution of differentially expressed lncRNAs at the GO biological process (BP), cellular component (CC) and molecular function (MF) levels (Figures 2b, 2c and 2d). Comprehensive bar plots from GO enrichment analysis showed that differentially expressed lncRNAs were mainly enriched in pathways related to immune response regulation, signal transduction, cell adhesion and transcriptional regulation (Figure 2e,f). Pathway statistics of upregulated lncRNA‐associated genes indicated predominant involvement in cell cycle and inflammatory response (Figure 2g). A heat map of differentially expressed lncRNAs clearly distinguished the TE and normal groups (Figure 2h). RT‐qPCR further validated that FENDRR expression was significantly increased in the TE group (Figure 2i,  ^∗∗∗^ *p* < 0.001).

Figure 2Bioinformatics analysis of microarray data comparing serum exosomes from healthy individuals (normal) and TEs (GSE213115). (a) Heat map of lncRNA expression. (b–d) GSEA of differentially expressed lncRNAs at the GO biological process (BP), cellular component (CC), and molecular function (MF) levels. (e) Barplot of GO enrichment analysis (BP, CC and MF). (f) Heat map of pathway‐level differences based on GSEA. (g) Enriched pathways associated with upregulated lncRNAs. (h) Heat map of differentially expressed lncRNAs. (i) RT‐qPCR validation of FENDRR expression in TEs. ∗∗∗*p* < 0.001.

**Figure:**
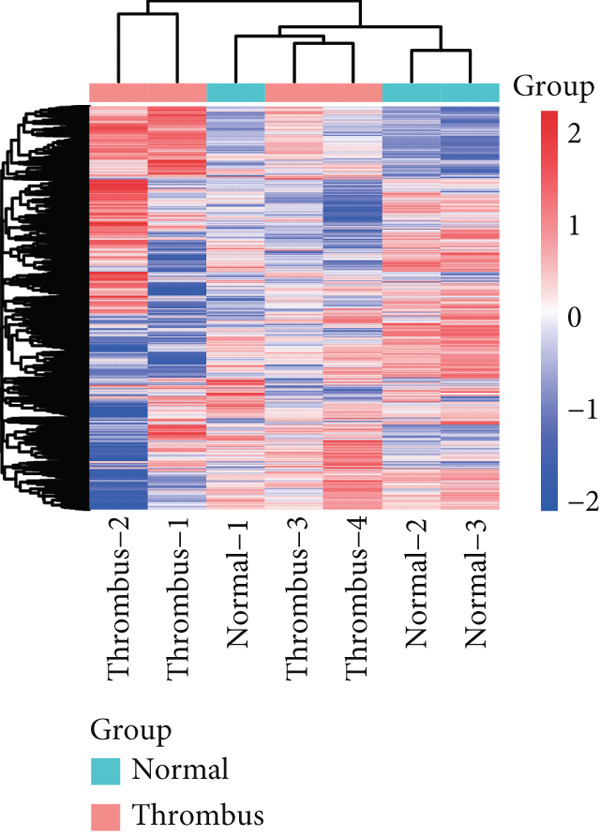
(a)

**Figure:**
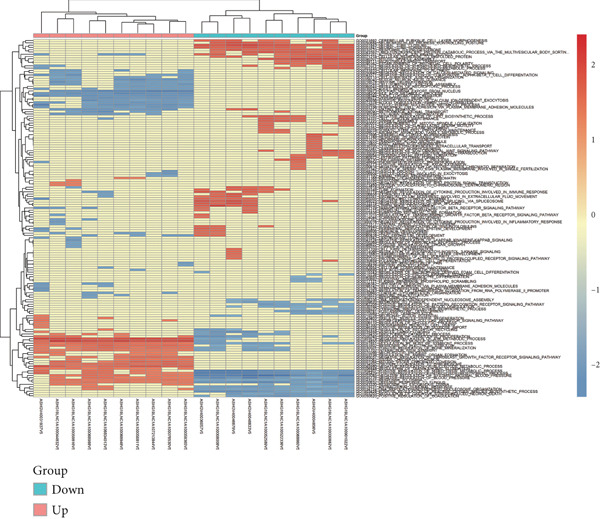
(b)

**Figure:**
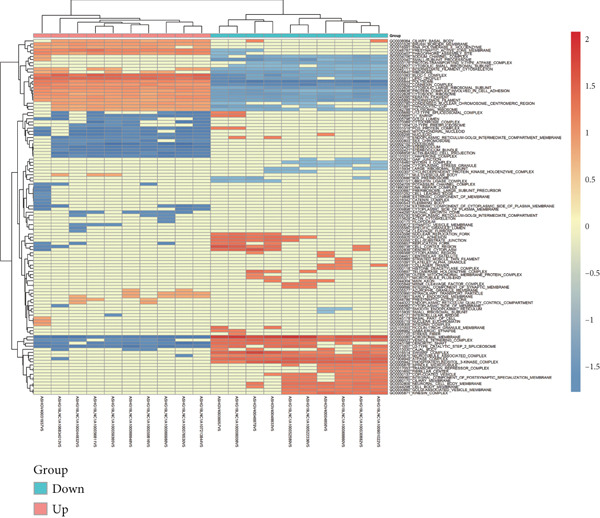
(c)

**Figure:**
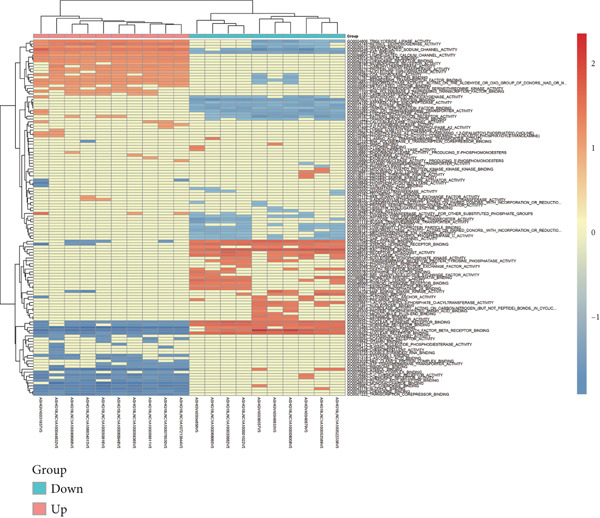
(d)

**Figure:**
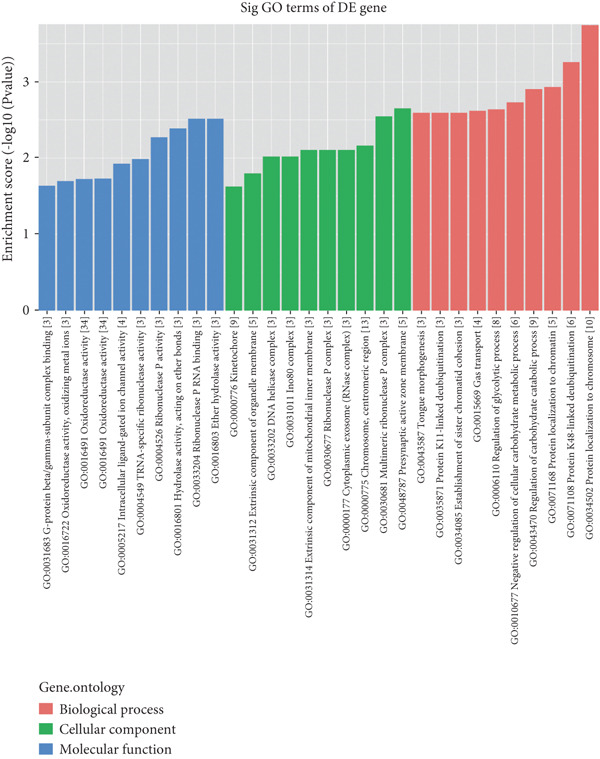
(e)

**Figure:**
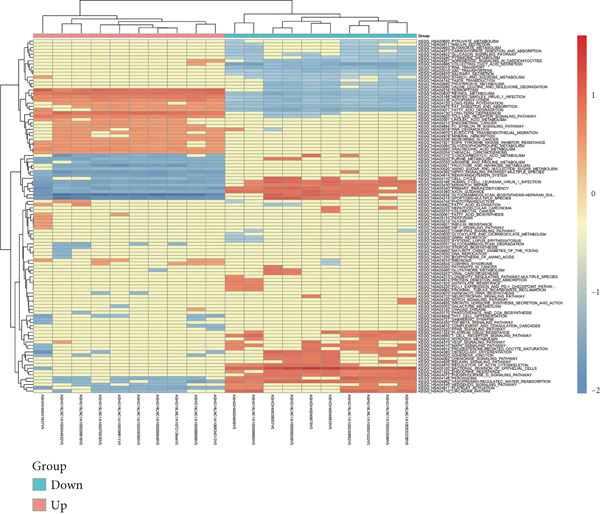
(f)

**Figure:**
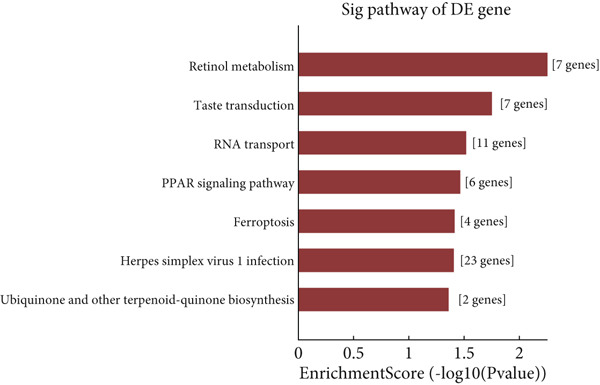
(g)

**Figure:**
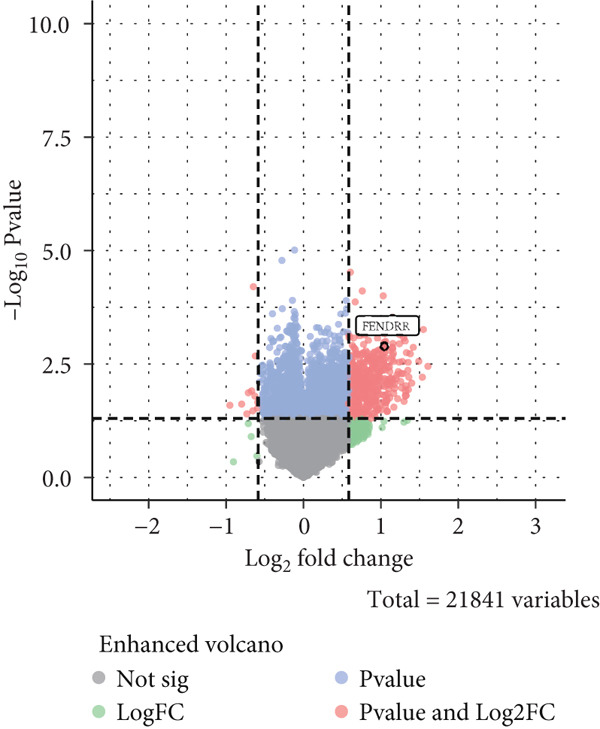
(h)

**Figure:**
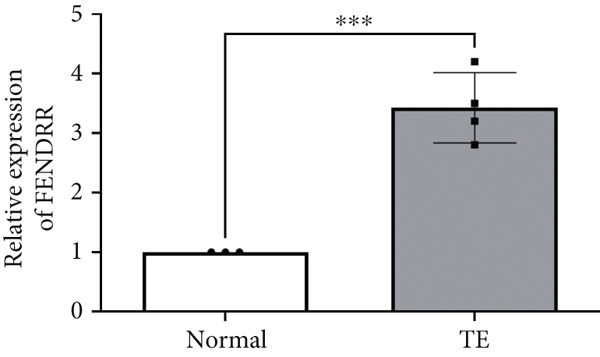
(i)

### 3.3. Regulatory Role of FENDRR in Cardiomyocyte Models

To further elucidate the function of FENDRR in cardiomyocytes, we constructed models with silenced (si‐FENDRR) or overexpressed FENDRR (OE‐FENDRR), as well as co‐cultures of TEs with AC16 cardiomyocytes. As shown in Figure 3a, TE treatment significantly reduced cell viability compared with the control group (*p* < 0.05), and FENDRR overexpression produced a similar effect (*p* < 0.05). Compared with TE alone, TE plus si‐FENDRR intervention resulted in improved cell viability (*p* < 0.05). Flow cytometry indicated that TE treatment increased the proportion of apoptotic cells (*p* < 0.05), whereas si‐FENDRR attenuated apoptosis compared with TE alone (*p* < 0.05), and OE‐FENDRR further promoted apoptosis (*p* < 0.05) (Figure 3b,c).

Figure 3Functional validation of FENDRR in cardiomyocytes (AC16 cells). (a) CCK8 assay for cell viability across experimental groups. (b) Flow cytometry for apoptosis (Annexin V and PI staining). (c) Statistical analysis of apoptosis rates. (d) Flow cytometry validation of DCFH‐DA staining for ROS. (e) Statistical analysis of ROS fluorescence intensity. (f) Western blot detection of key ferroptosis and ferritinophagy proteins: NCOA4, GPX4 and P62. (g–i) Quantification of protein expression levels for NCOA4 (g), GPX4 (h) and P62 (i). (j–l) qRT‐PCR analysis of mRNA expression levels for NCOA4 (j), GPX4 (k) and P62 (l). ns, not significant;  ^∗^ *p* < 0.05;  ^∗∗^ *p* < 0.01; ∗∗∗*p* < 0.001;  ^∗∗∗∗^ *p* < 0.0001.

**Figure:**
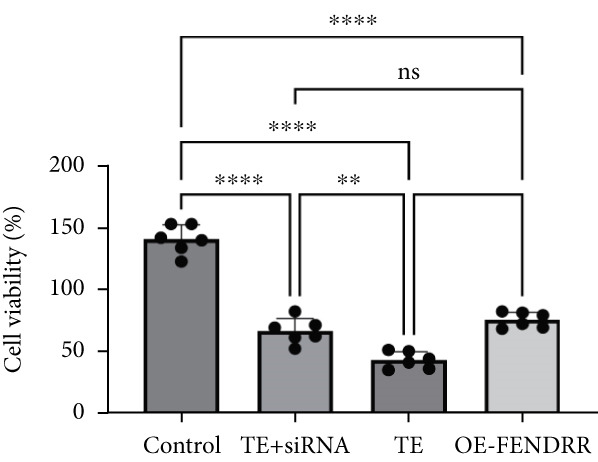
(a)

**Figure:**
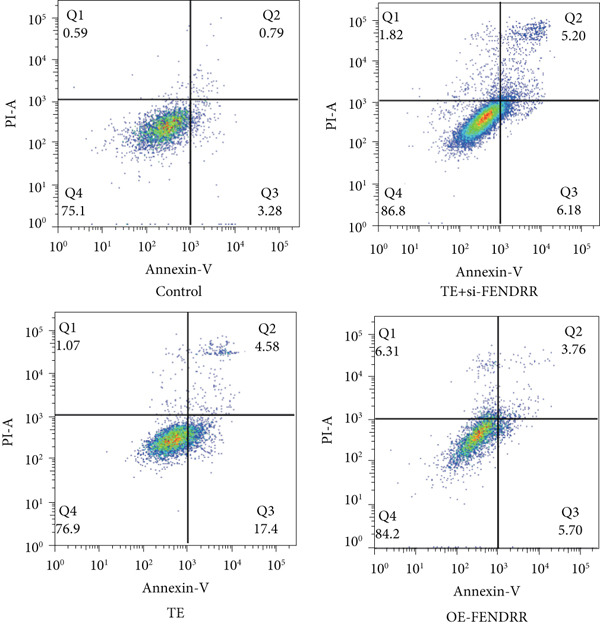
(b)

**Figure:**
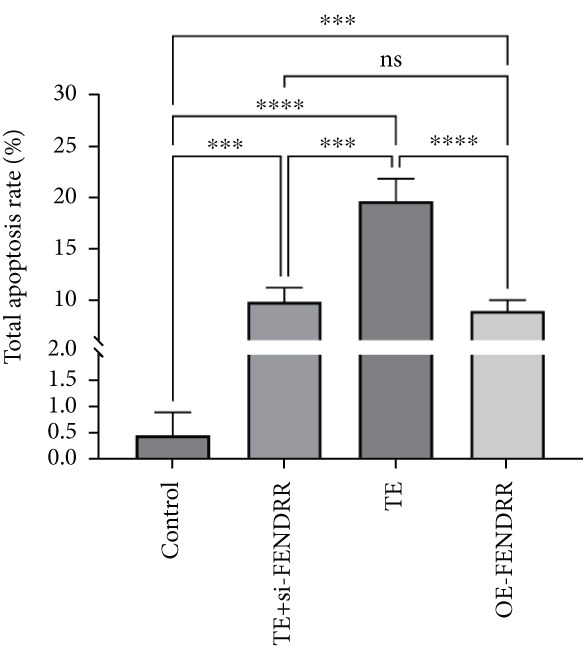
(c)

**Figure:**
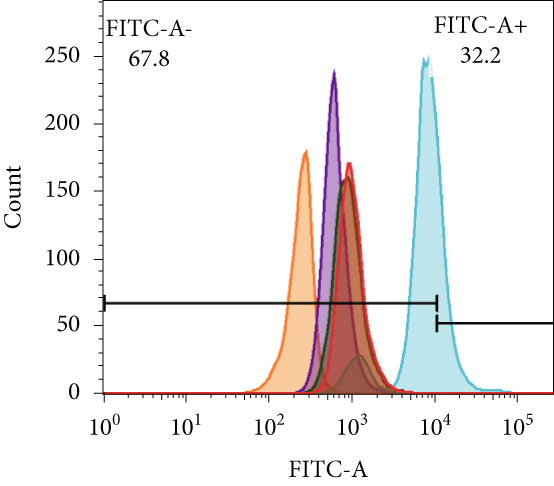
(d)

**Figure:**
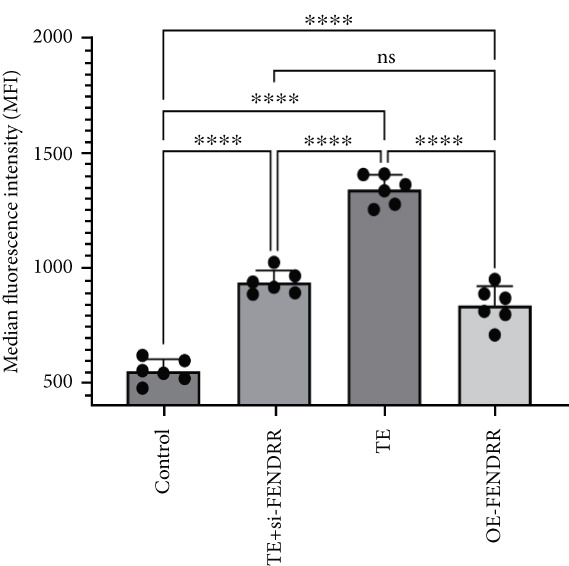
(e)

**Figure:**
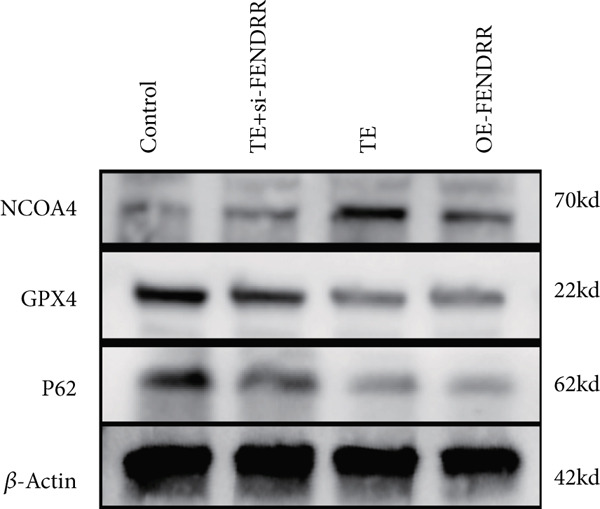
(f)

**Figure:**
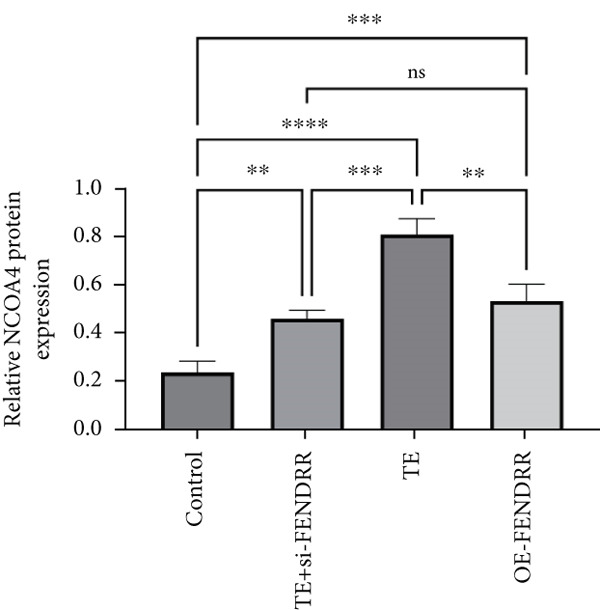
(g)

**Figure:**
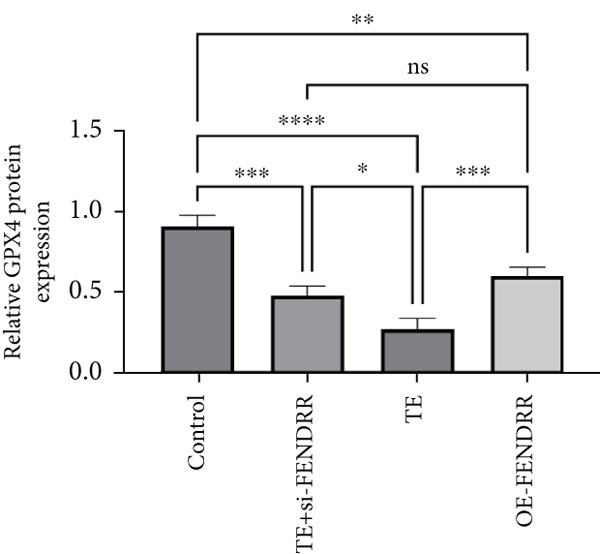
(h)

**Figure:**
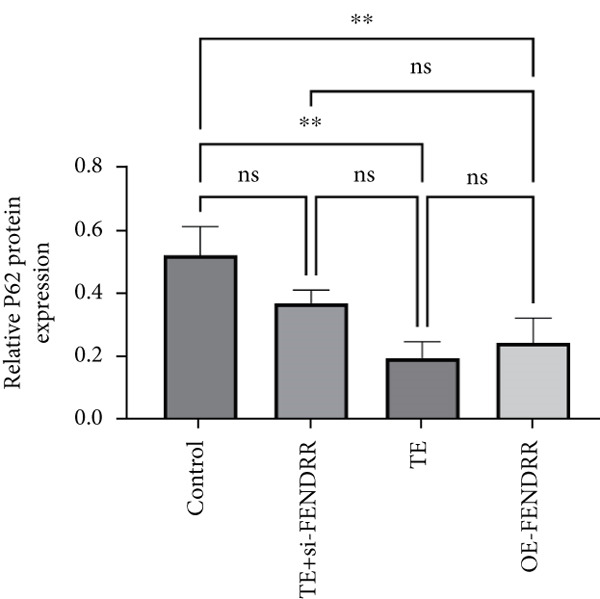
(i)

**Figure:**
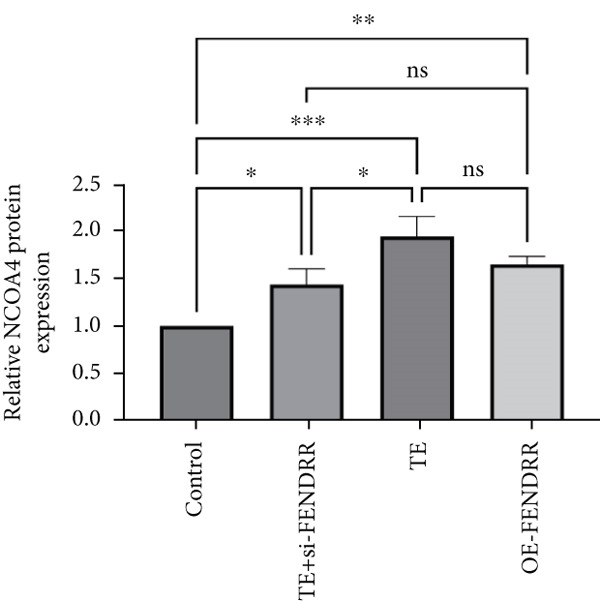
(j)

**Figure:**
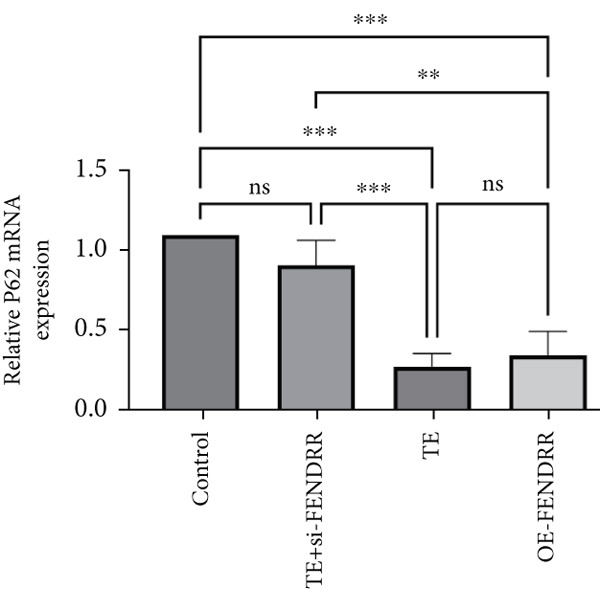
(k)

**Figure:**
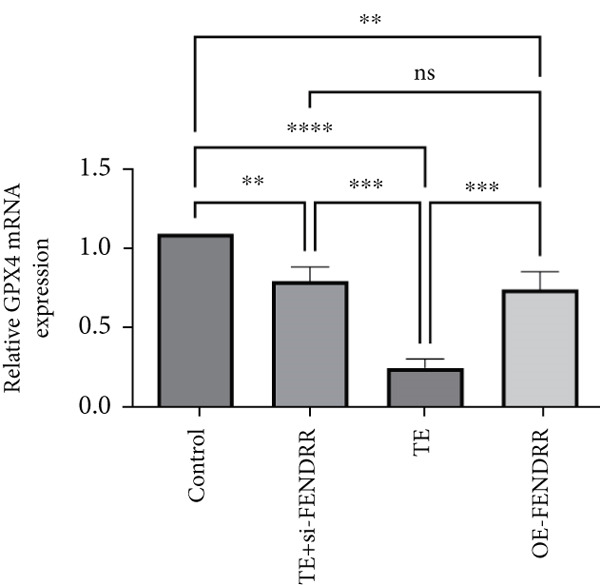
(l)

DCFH‐DA staining and flow cytometry (Figure 3d,e) demonstrated that TE stimulation significantly increased intracellular ROS levels (*p* < 0.05), which was ameliorated by si‐FENDRR (*p* < 0.05). Protein analyses showed that NCOA4 was upregulated and GPX4 and P62 were downregulated in the TE and OE‐FENDRR groups (*p* < 0.05), whereas si‐FENDRR significantly reversed these effects (*p* < 0.05) (Figures 3f, 3g, 3h and 3i). These findings were supported at the mRNA level (Figures 3j, 3k and 3l). Collectively, these results indicate that TEs promote ferroptosis and ferritinophagy in cardiomyocytes, with FENDRR as a key mediator.

### 3.4. Regulatory Effect of FENDRR on Myocardial Injury in the T1MI Animal Model

In the T1MI rat model, HE and Masson′s staining demonstrated marked myocardial injury and fibrosis in the T1MI group, which was alleviated by shFENDRR intervention (Figure 4a). Immunohistochemistry and qPCR revealed that, compared with the sham group, the T1MI group exhibited significantly increased NCOA4 expression at both protein (Figures 4b, 4c, 4d and 4e) and mRNA levels (Figures 4f, 4g and h), alongside decreased GPX4 and P62 expression (*p* < 0.05). These changes were ameliorated by shFENDRR treatment (*p* < 0.05). Additionally, ferroptosis and ferritinophagy markers Fe^2+^ and MDA were elevated in the T1MI group, whereas SOD activity was reduced (Figures 4i, 4j and 4k); shFENDRR treatment significantly improved these indices (*p* < 0.05). These results suggest that T1MI‐induced myocardial injury may be mediated by altered FENDRR expression, with mechanisms closely related to ferroptosis and ferritinophagy regulation.

Figure 4Animal experiments validating the effect of FENDRR on myocardial injury in the T1MI model (*n* = 6). (a) HE and Masson staining for each experimental group. (b) Immunohistochemical detection of NCOA4, GPX4 and P62. (c–e) Quantification of immunohistochemical staining for NCOA4 (c), P62 (d) and GPX4 (e). (f–h) qRT‐PCR analysis of mRNA expression for NCOA4 (f), P62 (g) and GPX4 (h). (i–k) Quantification of Fe^2+^ (i), MDA (j) and SOD (k) expression in myocardial tissue.  ^∗^ *p* < 0.05;  ^∗∗^ *p* < 0.01;  ^∗∗∗^ *p* < 0.001;  ^∗∗∗∗^ *p* < 0.0001.

**Figure:**
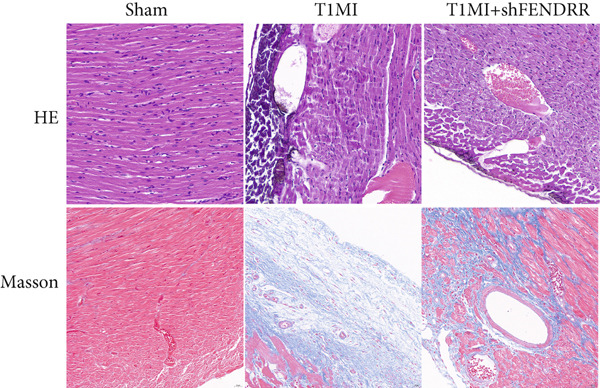
(a)

**Figure:**
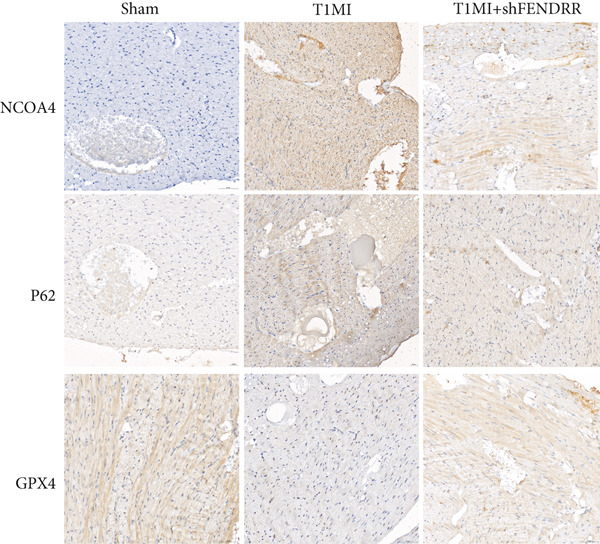
(b)

**Figure:**
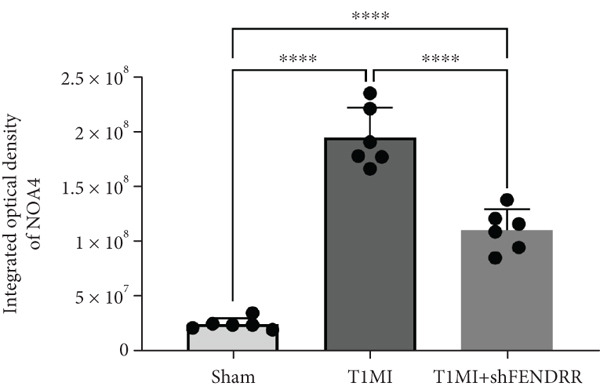
(c)

**Figure:**
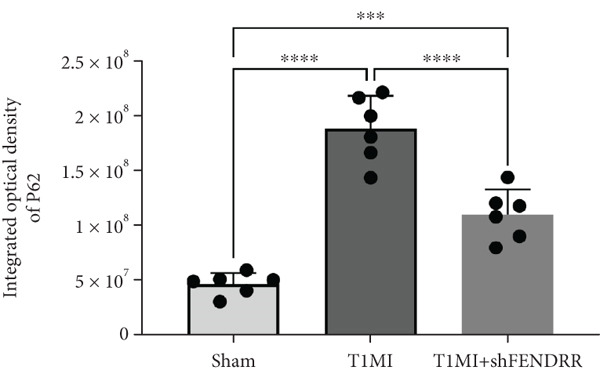
(d)

**Figure:**
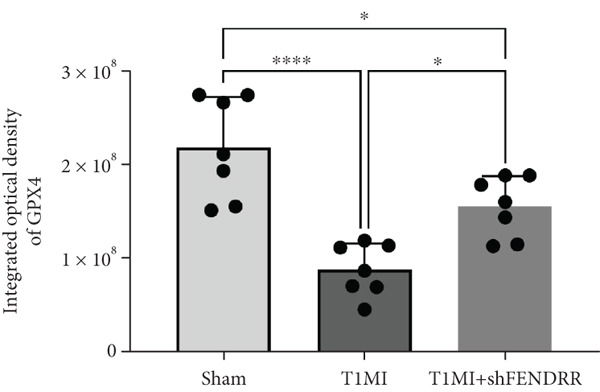
(e)

**Figure:**
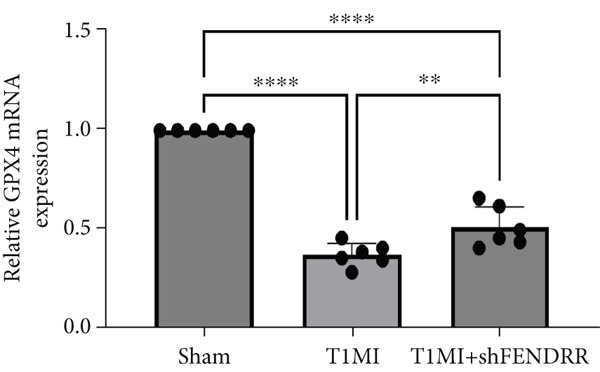
(f)

**Figure:**
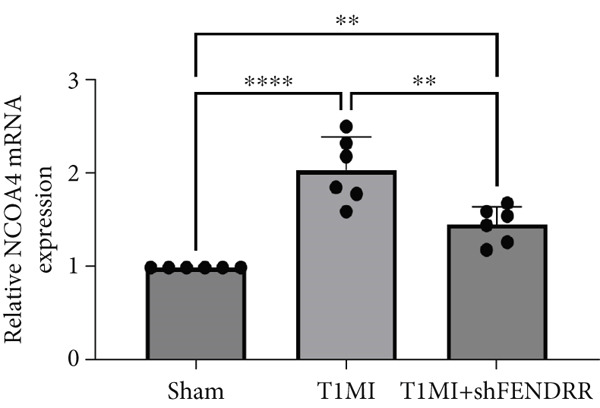
(g)

**Figure:**
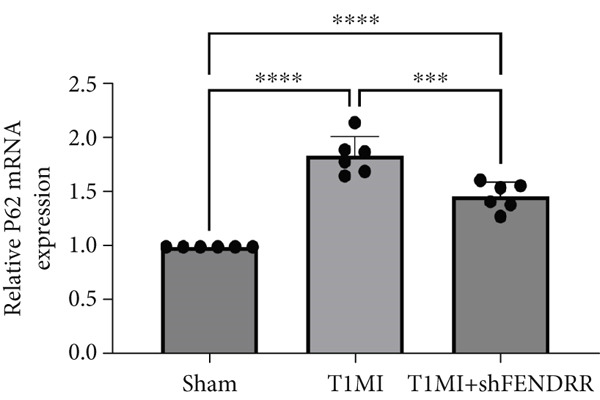
(h)

**Figure:**
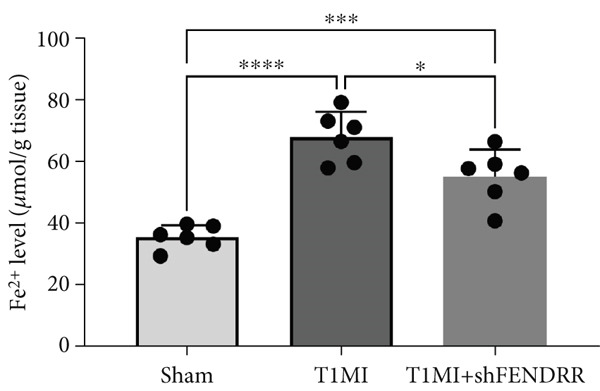
(i)

**Figure:**
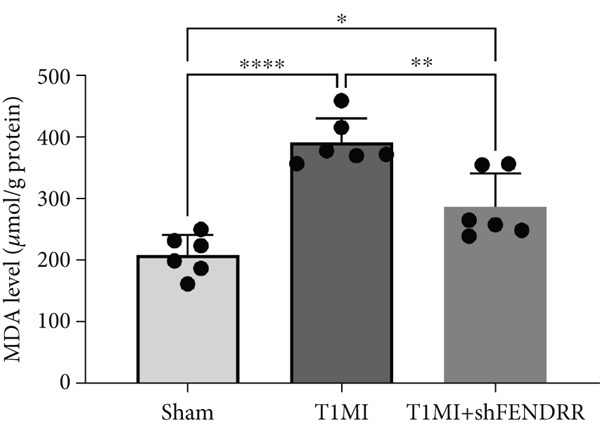
(j)

**Figure:**
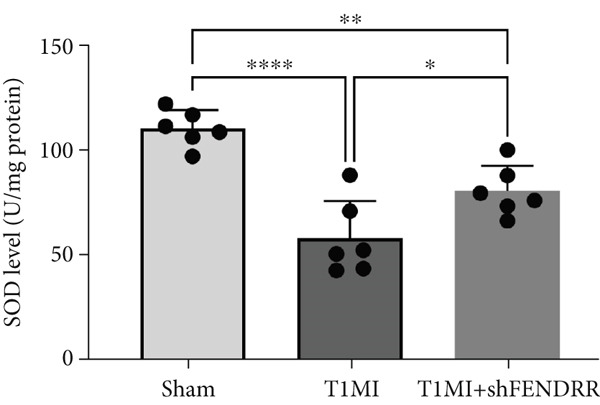
(k)

### 3.5. Expression Pattern and Prognostic Significance of FENDRR in Human Cancers

After elucidating the role of FENDRR in T1MI, we next examined its expression landscape across human cancers and its clinical implications. Pan‐cancer analyses revealed that FENDRR was consistently downregulated in several solid tumours compared with normal tissues, with an especially marked reduction in LUAD (Figure 5a). This trend was further confirmed when tumour tissues were compared with nontumour controls (Figure 5b), as well as through paired analyses of tumour and adjacent tissues (Figure 5c).

Figure 5Expression pattern and prognostic value of FENDRR in human cancers. (a) Differential expression of FENDRR across multiple cancer types compared with normal tissues. (b) Comparison of FENDRR levels between tumour and non‐tumour tissues. (c) Paired analysis of FENDRR expression in tumour samples and their matched adjacent tissues. (d) Time‐dependent ROC curves assessing the predictive performance of FENDRR for overall survival in LUAD. ns, not significant;  ^∗^ *p* < 0.05;  ^∗∗^ *p* < 0.01;  ^∗∗∗^ *p* < 0.001.

**Figure:**
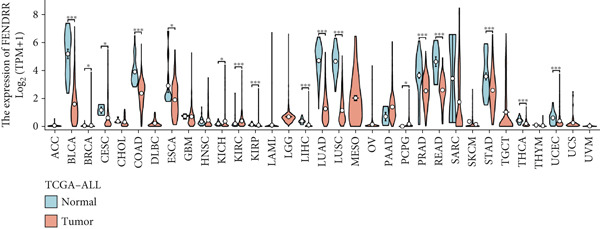
(a)

**Figure:**
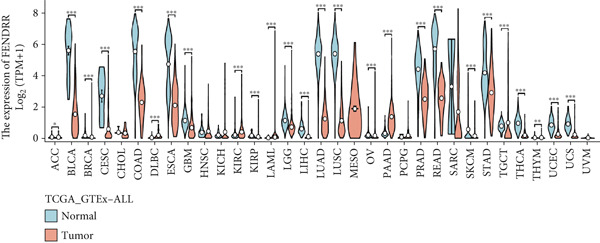
(b)

**Figure:**
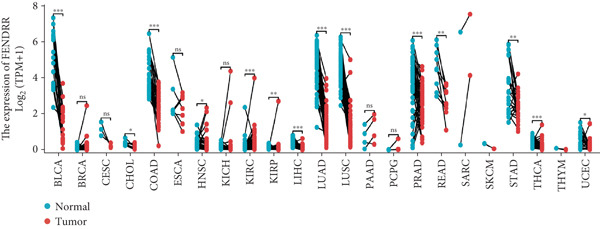
(c)

**Figure:**
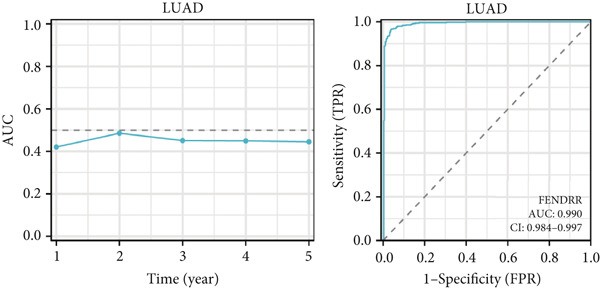
(d)

Given these expression patterns, we assessed the prognostic relevance of FENDRR in LUAD. Time‐dependent ROC analysis demonstrated excellent predictive performance, with a 5‐year AUC of 0.990 (95% CI: 0.984–0.997), and similarly high discrimination across earlier time points (Figure 5d). These results indicate that the downregulation of FENDRR in lung cancer is not only a disease‐specific molecular feature but may also hold significant prognostic value.

### 3.6. Immune Cell Infiltration Landscape Associated With FENDRR Expression

Building on its expression pattern in LUAD, we further explored how FENDRR relates to immune cell infiltration within the tumour microenvironment. CIBERSORT analysis demonstrated broad associations between FENDRR expression and several immune cell subsets, including CD8^+^ T cells, NK cells and M1/M2 macrophages (Figure 6a). ssGSEA produced similar results, showing consistent correlations across multiple immune infiltration signatures (Figure 6b).

Figure 6Associations between FENDRR expression and immune cell infiltration. (a) Correlation between FENDRR and immune cell subsets estimated by the CIBERSORT algorithm. (b) ssGSEA‐based heat map showing relationships between FENDRR expression and immune infiltration signatures. (c) Spearman correlation matrix integrating seven immune deconvolution algorithms. (d) Differences in major immune cell populations between high‐ and low‐FENDRR groups. (e) Comparison of stromal, immune, and estimate scores across FENDRR expression groups. (f) Lollipop plot summarizing the multi‐algorithm associations between FENDRR and the tumour immune microenvironment. *p < 0.05.

**Figure:**
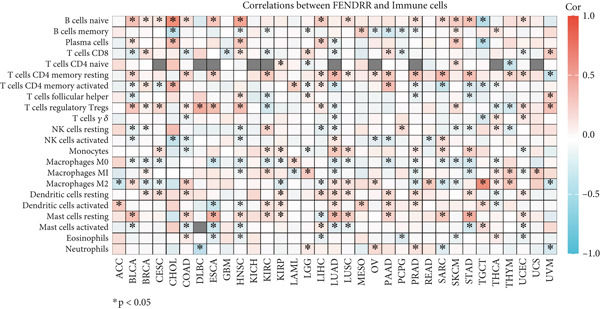
(a)

**Figure:**
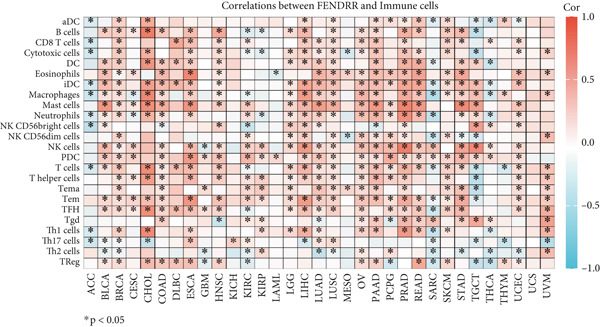
(b)

**Figure:**
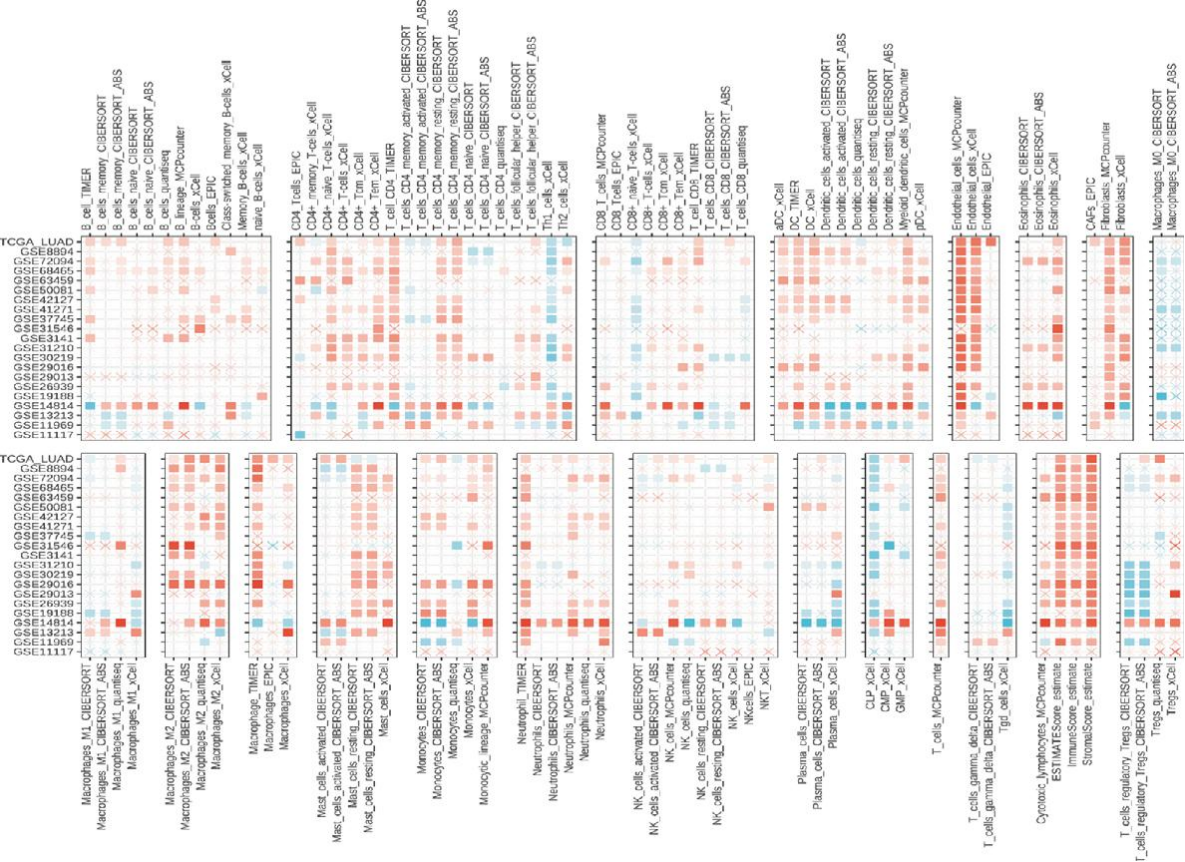
(c)

**Figure:**
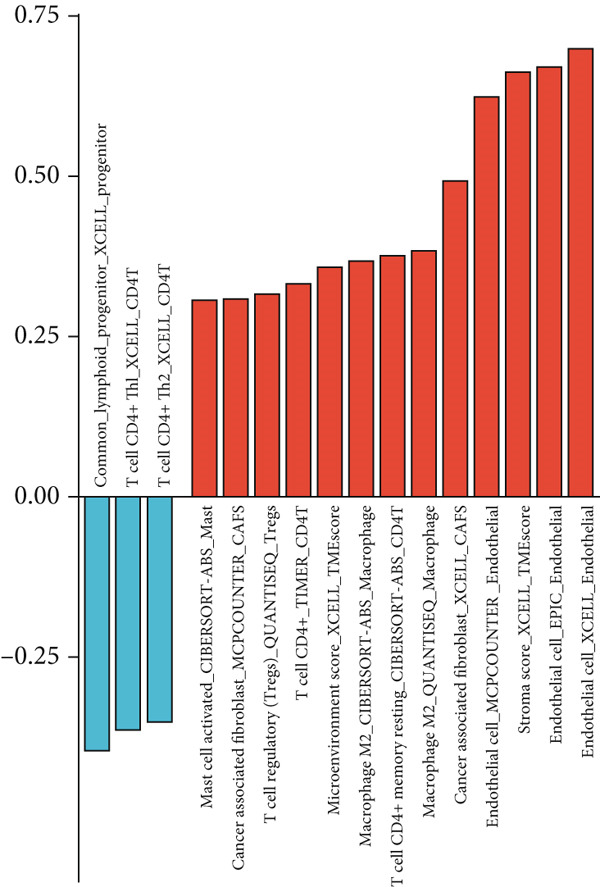
(d)

**Figure:**
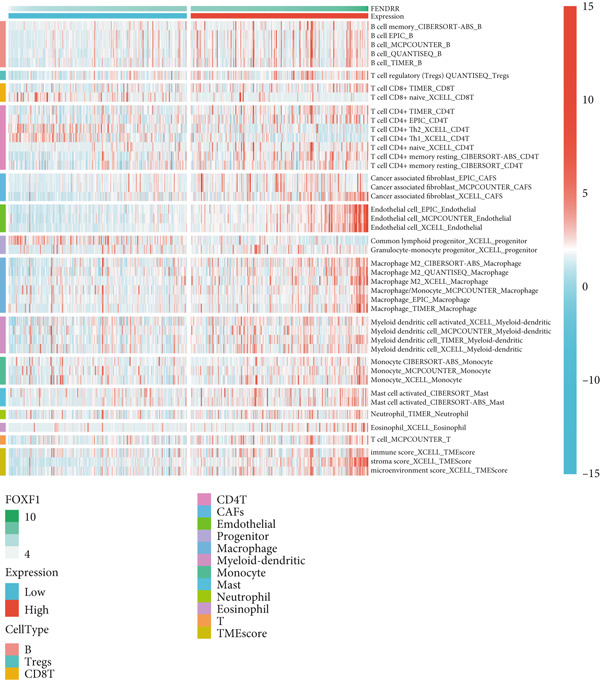
(e)

**Figure:**
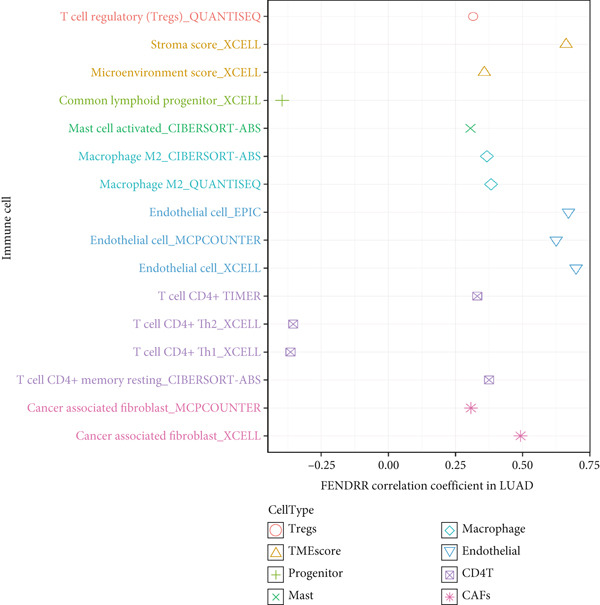
(f)

To strengthen these observations, we incorporated additional immune deconvolution approaches. Correlation analyses revealed that the overall trends were comparable across these algorithms (Figure 6c). When immune cell proportions were compared between the high‐ and low‐FENDRR groups (Figure 6d), several major immune cell populations—including cytotoxic T cells and inflammation‐related macrophages—displayed significant differences. In parallel, immune, stromal and estimate scores were all elevated in the high‐expression group (Figure 6e), suggesting that FENDRR expression is closely linked to the overall activity of the tumour microenvironment. An integrated lollipop analysis summarized these multialgorithm findings and further highlighted the systemic relationship between FENDRR and immune infiltration profiles (Figure 6f).

### 3.7. Immune Activation Signatures and Regulatory Features Related to FENDRR

To complement the infiltration analyses, we evaluated whether FENDRR expression was associated with distinct immune activation states. TIP analysis revealed that higher FENDRR levels were linked to more active immune response processes (Figure 7a). Consistently, MeTIL, T cell‐inflamed and TLS scores were all significantly elevated in the high‐expression group (Figures 7b, 7c and 7d), suggesting that FENDRR may contribute to the development of anti‐tumour immune structures and effector responses.

Figure 7Immune activity, regulatory features and genomic context associated with FENDRR. (a) TIP analysis illustrating immune activation patterns in relation to FENDRR expression. (b) Comparison of MeTIL scores between high‐ and low‐FENDRR groups. (c) T cell‐inflamed score stratified by FENDRR expression. (d) Differences in tertiary lymphoid structure (TLS) scores associated with FENDRR levels. (e) Expression of key immune regulatory genes across FENDRR groups. (f) Heat map of immune‐stimulatory, immune‐suppressive, chemokine and HLA gene expression patterns. (g) Genomic and antigen‐presentation characteristics in relation to FENDRR expression.

**Figure:**
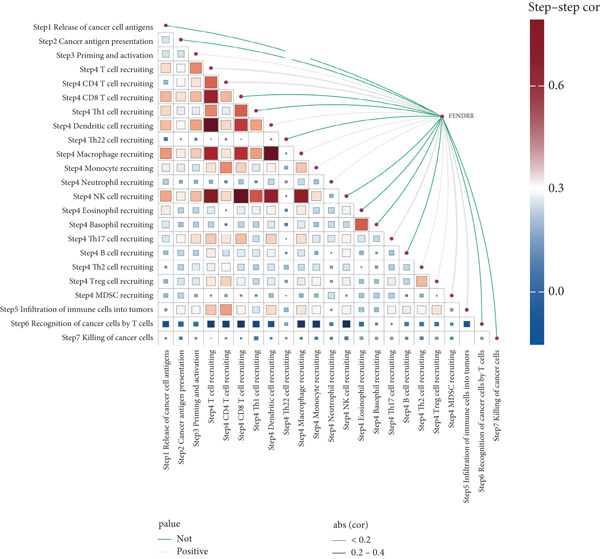
(a)

**Figure:**
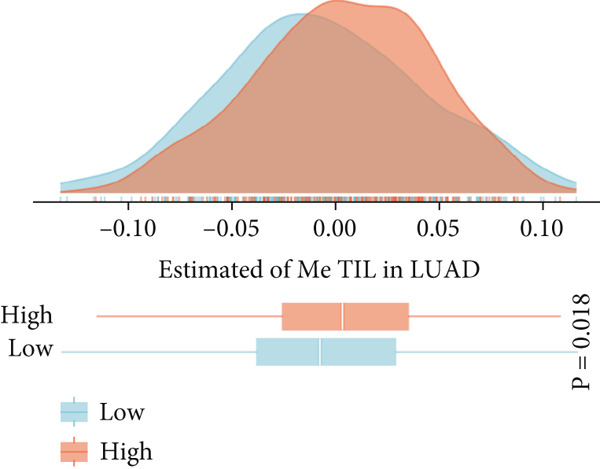
(b)

**Figure:**
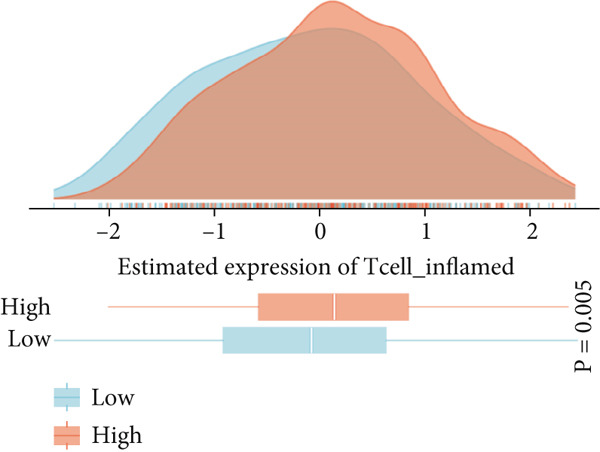
(c)

**Figure:**
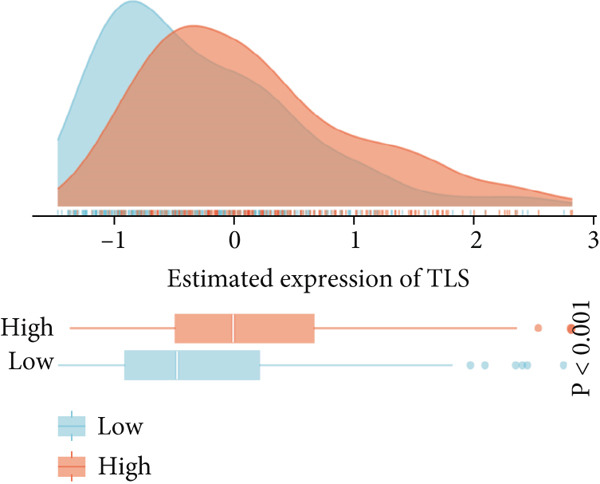
(d)

**Figure:**
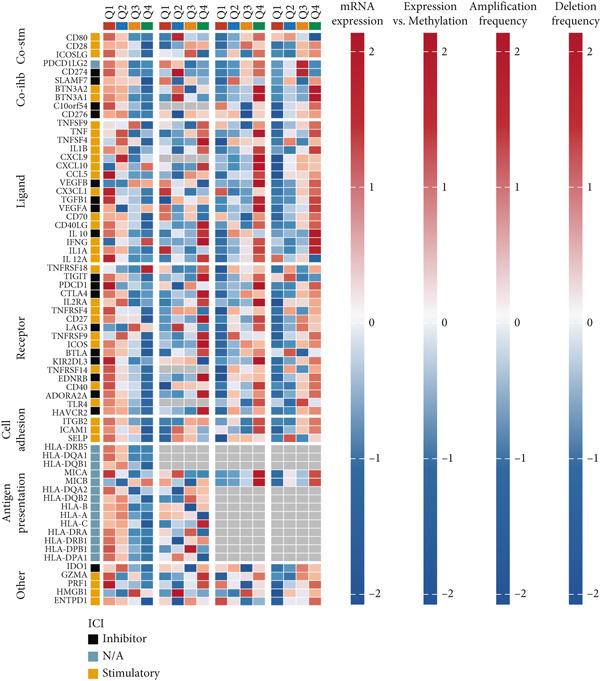
(e)

**Figure:**
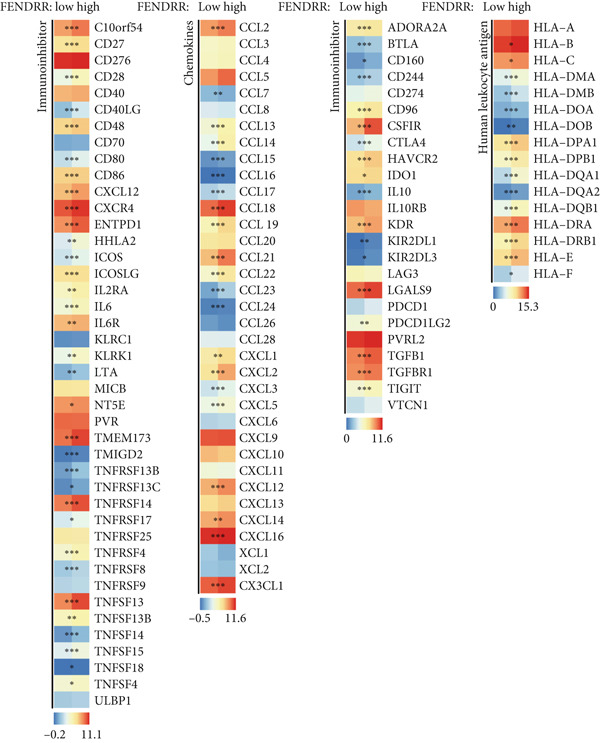
(f)

**Figure:**
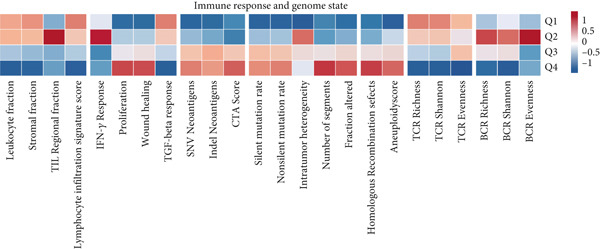
(g)

At the regulatory level, higher FENDRR expression coincided with increased levels of multiple immune‐related gene categories, including immune‐stimulatory and immune‐suppressive regulators, chemokines and HLA genes (Figures 7e,f). These changes were accompanied by enhanced antigen‐presentation capacity and genomic features associated with immune responsiveness (Figure 7g), indicating a broader immunological influence of FENDRR within the tumour context.

Because immune‐related signatures may display varying degrees of sensitivity, we included additional analyses of chemokine signatures, cytolytic activity and IFN*γ* responses in the supporting information (Figures S1a, S1b and S1c). Although these indices did not show the same magnitude of difference as the primary immune activation scores, their overall patterns still supported a link between FENDRR expression and immune functional activity.

### 3.8. Omics Lineage, Machine Learning Modelling and Mechanistic Network of FENDRR‐Related Molecules

In representative tumours such as LUAD, differential analysis between high and low FENDRR expression groups identified significant enrichment of gene pathways associated with cell cycle, inflammation and signal transduction (Figure S2a,b). Further COX regression analysis (Figure S2c) pinpointed key prognostic genes including KCNQ1‐AS1 (HR = 1.443, *p* = 0.021) and APAP1 (HR = 1.312, *p* < 0.001), which act as risk factors and together with FENDRR constitute a unique regulatory network.

To assess the prognostic utility of FENDRR‐related genes, a prognostic model based on FENDRR risk scores was constructed using Lasso, CoxBoost, Ridge and other machine learning algorithms. Cross‐validation across multiple cohorts showed excellent performance in both TCGA and GEO datasets (C − index = 0.71, Figure 8a). In the test and training sets, the AUCs for 1‐, 3‐ and 5‐year survival were 0.862, 0.830, 0.825 and 0.706, 0.696, 0.63, respectively (Figure 8b,c). Kaplan–Meier analysis indicated sustained poorer survival in the high‐risk group (*p* < 0.001, Figure 8d,e). The multi‐year c‐index comparison produced similar conclusions (C − index = 0.715, Figure 8f), indicating that this risk model is an independent prognostic factor (Figure 8g). Incorporating clinical features into a nomogram further enhanced the predictive power for individual survival (Figure 8h).

Figure 8Prognostic analysis of FENDRR in LUAD using machine learning models. (a) C‐index heat map for machine learning models. (b) Time‐dependent AUC in the test set. (c) Time‐dependent AUC in the training set. (d) Survival curves in the test set. (e) Survival curves in the training set. (f) Line plot of C‐index across multiple years. (g) Comparison of C‐index for multivariate models. (h) Nomogram incorporating clinical features.

**Figure:**
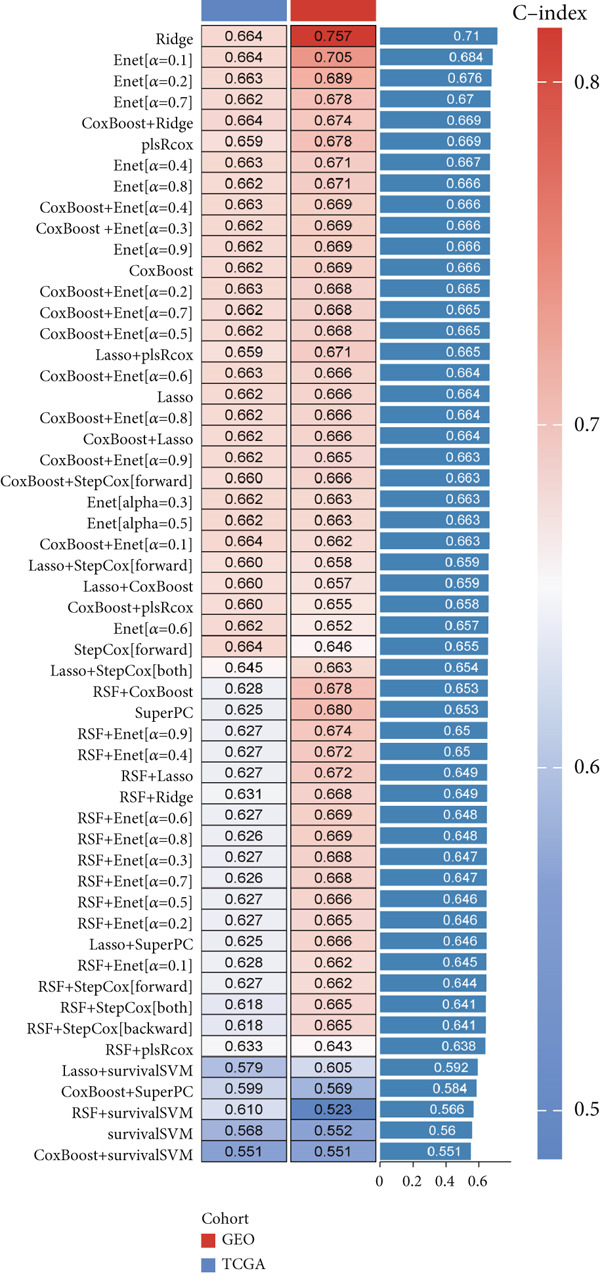
(a)

**Figure:**
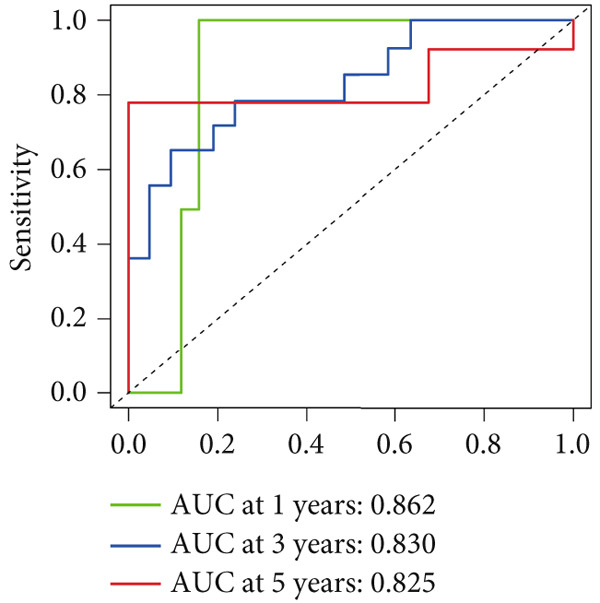
(b)

**Figure:**
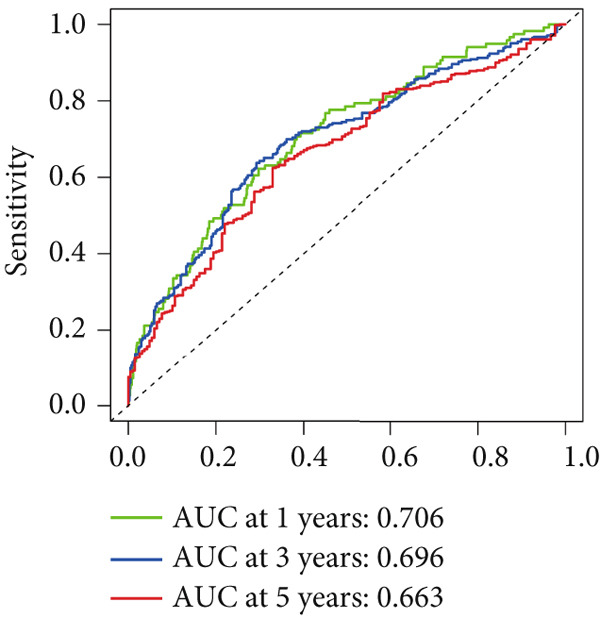
(c)

**Figure:**
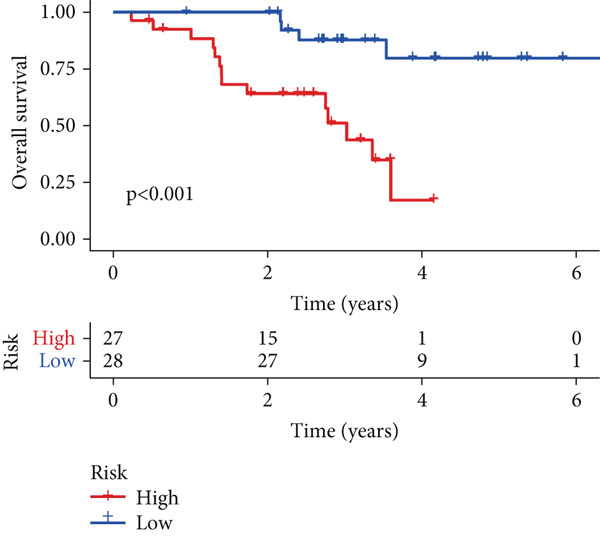
(d)

**Figure:**
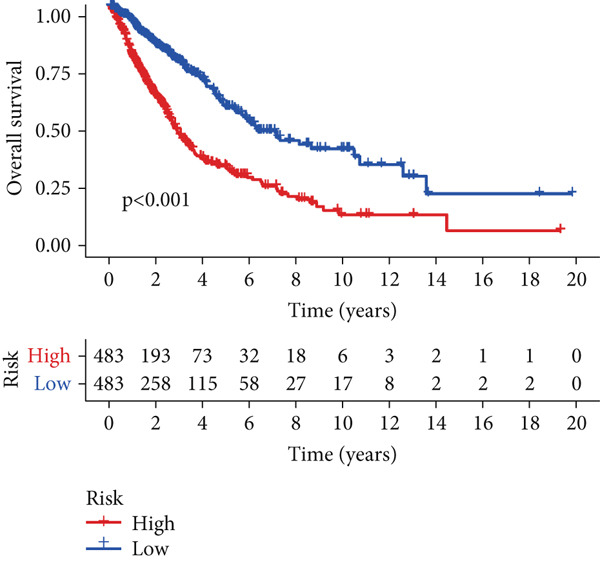
(e)

**Figure:**
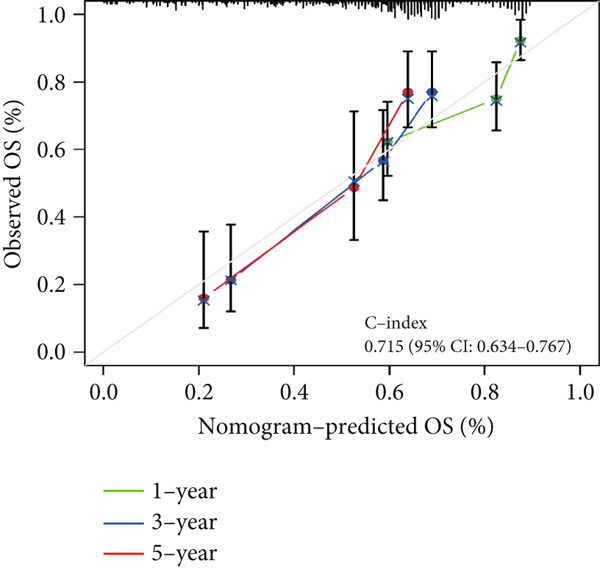
(f)

**Figure:**
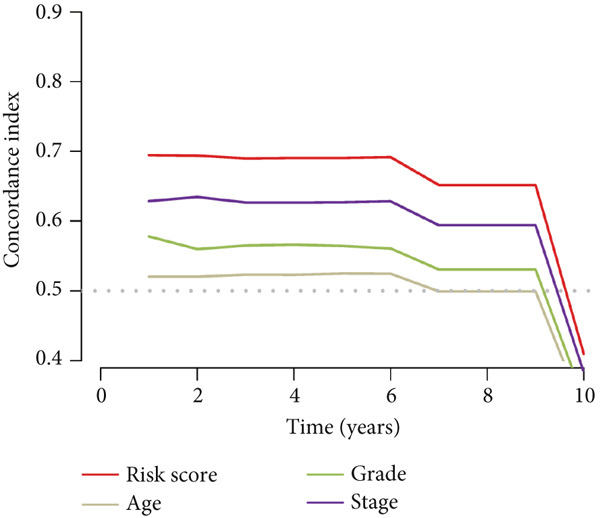
(g)

**Figure:**
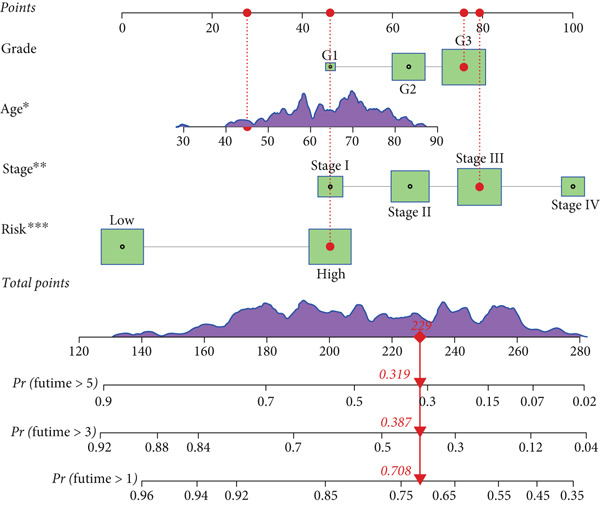
(h)

STRING protein–protein interaction network analysis revealed a high degree of coordination among downstream FENDRR‐regulated genes (Figures 9a, 9b and 9c). Interaction clusters represented by CXCL8, NT5E and KREMEN2 not only exhibited close genomic proximity (Figure 9b) but also showed diagnostic AUCs exceeding 0.5, with PTX3 reaching 0.946 (Figure 9d). Time‐dependent AUCs at various survival points were all above 0.60 (Figure 9e), suggesting robust diagnostic and long‐term prognostic potential. This combined omics and machine learning approach greatly expands the application landscape of FENDRR as a molecular marker.

Figure 9Protein interaction, gene localisation and prognostic analysis of FENDRR‐related genes. (a) Protein interaction network. (b) Circos plot of chromosomal localisation for interacting genes. (c) Correlation plot of gene expression among interacting nodes. (d) ROC analysis for diagnostic value of interacting genes. (e) Time‐dependent AUC analysis for interacting genes.

**Figure:**
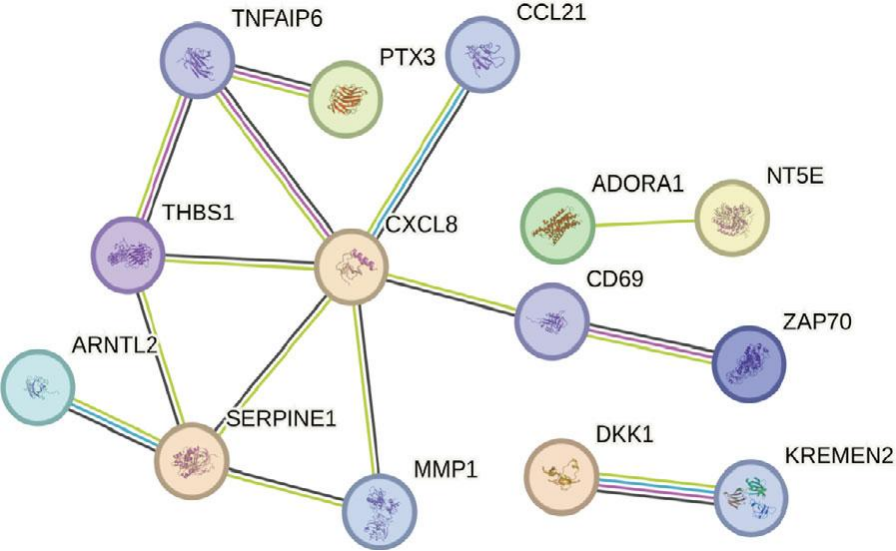
(a)

**Figure:**
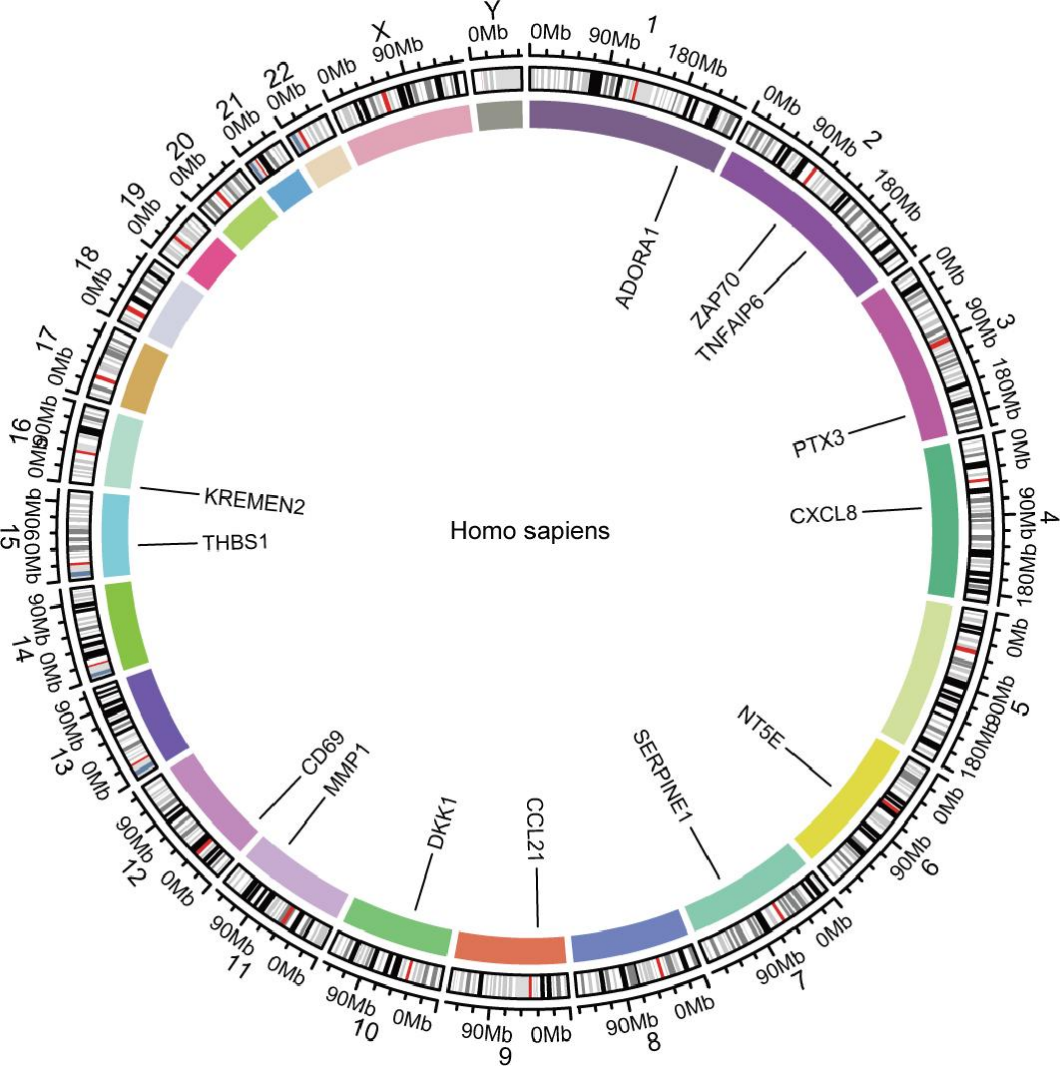
(b)

**Figure:**
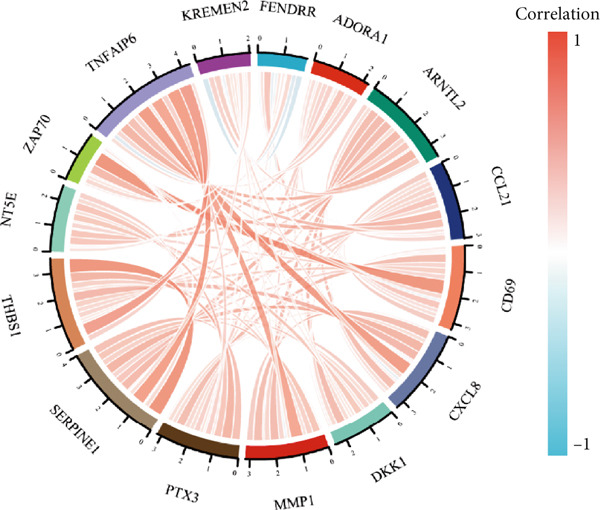
(c)

**Figure:**
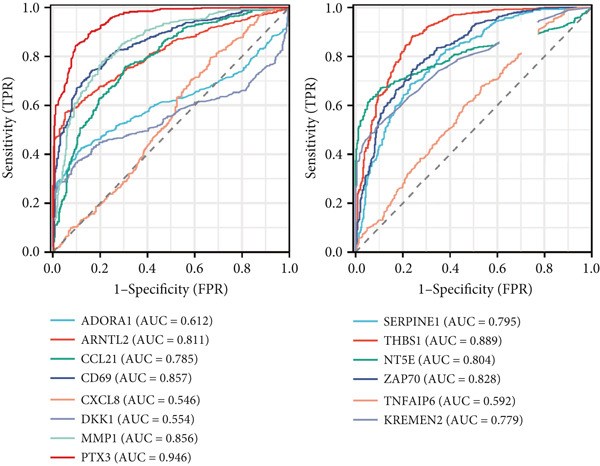
(d)

**Figure:**
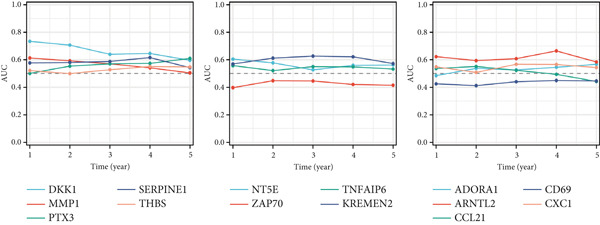
(e)

### 3.9. Single‐Cell Lineage Positioning and Regulatory Mechanisms

Single‐cell sequencing UMAP clustering (Figure 10a,b) indicated that FENDRR expression was higher in stromal and certain immune cell subsets than in malignant tumour cells (Figures 10c, 10d and 10e). This spatial and lineage‐specific localisation suggests that FENDRR may indirectly shape the tumour immune microenvironment and immune responses via stromal–immune signalling axes. These findings provide crucial single‐cell and multiomics evidence for FENDRR′s role as a molecular bridge in tumour–cardiovascular comorbidity.

Figure 10Single‐cell sequencing analysis. (a) UMAP of major cell lineages. (b) UMAP of FENDRR expression. (c) Expression differences of genes among immune, stromal and malignant lineages. (d) Mean gene expression in different main cell types. (e) Mean gene expression in detailed cell lineages.

**Figure:**
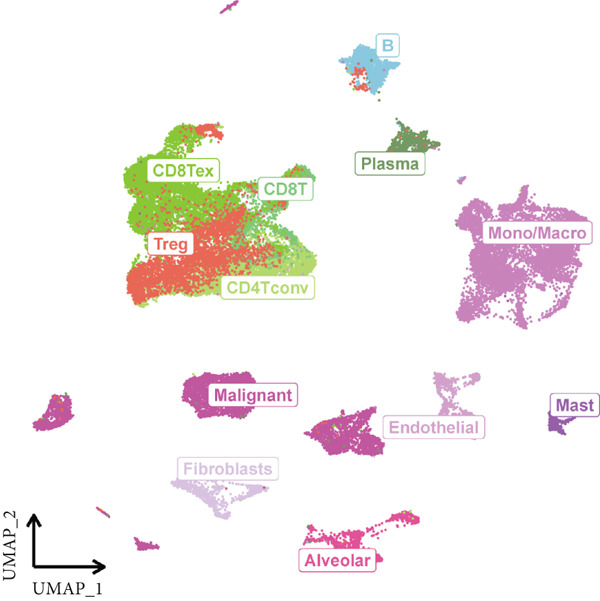
(a)

**Figure:**
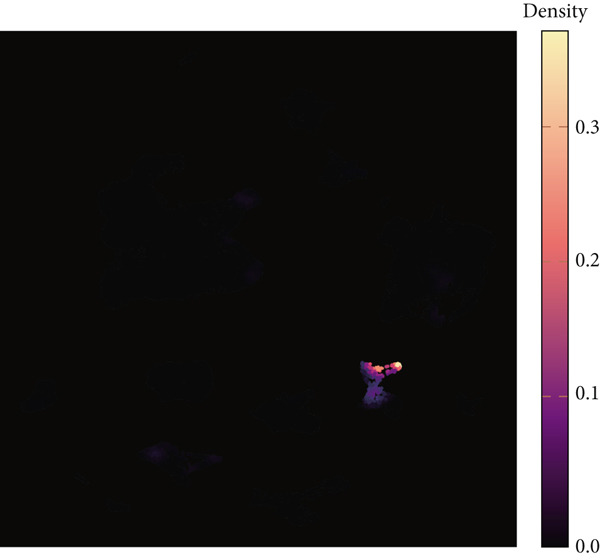
(b)

**Figure:**
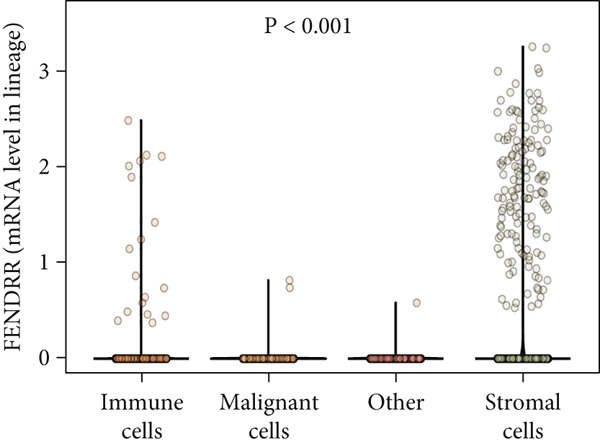
(c)

**Figure:**
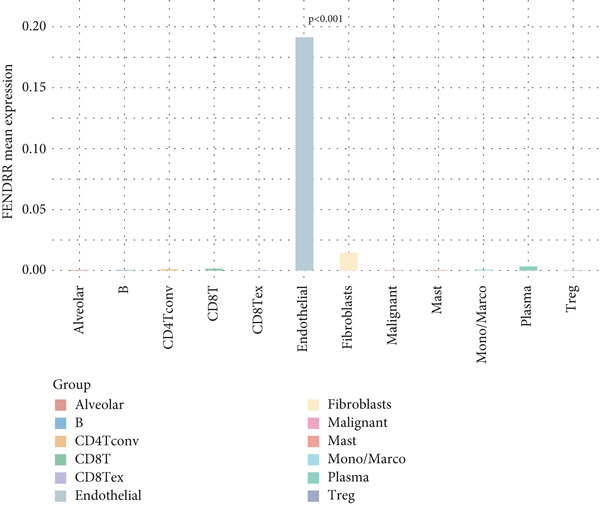
(d)

**Figure:**
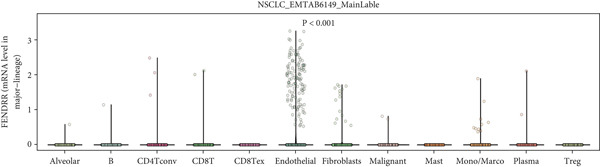
(e)

### 3.10. Immunohistochemical Features of Coronary Thrombi in Patients With Lung Cancer and T1MI

To further investigate how tumour background influences the immune microenvironment of myocardial infarction thrombi, we performed immunohistochemical analysis of coronary thrombus samples from the T1MI group and from patients with both LUAD and T1MI, assessing the expression levels of CD8, CXCL8, PTX3, PD‐L1 and FOXP3 (Figures 11a, 11b, 11c, 11d, 11e and 11f). The results showed that, compared with the T1MI group, the LUAD + T1MI group exhibited significantly increased expression of CD8 (Figure 11b), PTX3 (Figure 11d) and FOXP3 (Figure 11f) in coronary thrombi (*p* < 0.05), suggesting that a tumour background may enhance local T cell infiltration, inflammatory response and regulatory T cell (Treg) activity. However, the expression of CXCL8 (Figure 11c) and PD‐L1 (Figure 11e) did not differ significantly between groups (*p* > 0.05). These findings further support the notion that tumour‐specific factors can distinctively modulate the immune and inflammatory molecular profiles of coronary thrombi, potentially influencing the remodelling of the thrombus immune microenvironment and the development of heart–tumour interactions.

Figure 11Immunohistochemical analysis of key immune molecules in coronary thrombi from the T1MI group and the LUAD + T1MI group (*n* = 3). (a) Immunohistochemical staining of coronary thrombi for CD8 (b), CXCL8 (c), PTX3 (d), PD‐L1 (e) and FOXP3 (f), with statistical analysis of their expression levels.

**Figure:**
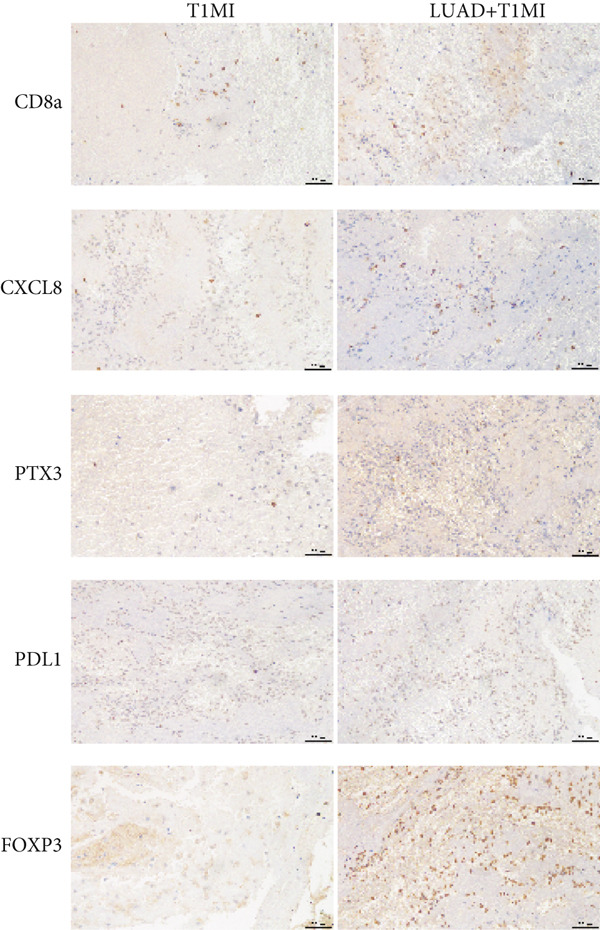
(a)

**Figure:**
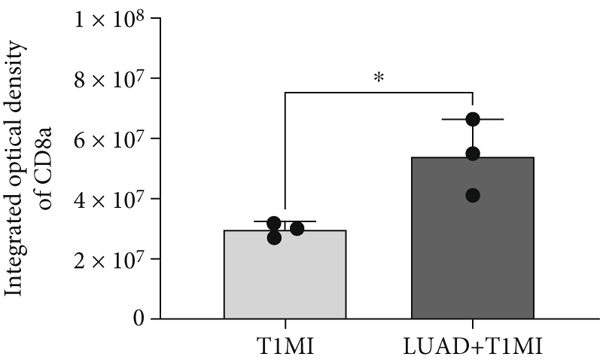
(b)

**Figure:**
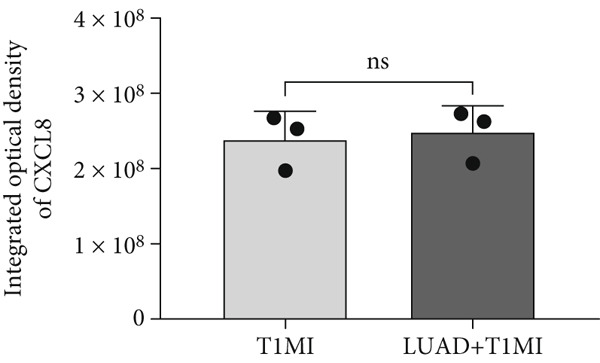
(c)

**Figure:**
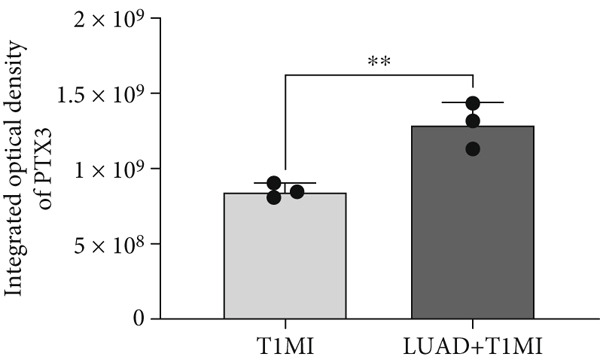
(d)

**Figure:**
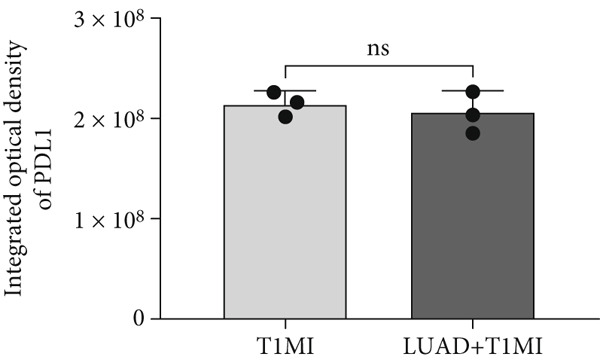
(e)

**Figure:**
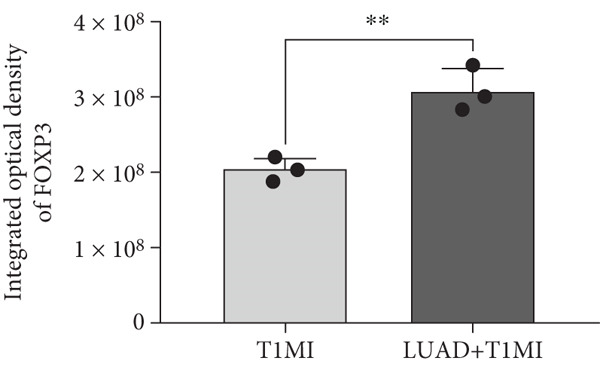
(f)

## 4. Discussion

This study, by integrating multiomics data, animal models, cellular functional assays and immunohistochemical analyses, systematically elucidates the potential causal relationship between lung cancer and T1MI and reveals the pivotal role of exosomal lncRNA FENDRR in the context of cancer–cardiovascular comorbidity. Our findings suggest that FENDRR is markedly upregulated in TEs from T1MI patients, where it promotes ferroptosis and immune remodelling, thereby exacerbating myocardial injury. In contrast, FENDRR is generally downregulated in lung cancer tissues, and higher FENDRR expression is associated with more favourable patient outcomes. This context‐dependent, tissue‐ and disease‐specific functional divergence represents a frontier question in the intersection of oncology and cardiovascular research [22], offering new perspectives for mechanistic studies and precision interventions.

To further place these findings in context, it is worth noting that T1MI is not merely a localized ischemic event but a systemic inflammatory condition in which circulating exosomes actively participate in inter‐organ communication [7–9]. Our observation that TEs carry markedly elevated FENDRR suggests that cardiomyocyte injury may be amplified by signals originating from the thrombotic microenvironment itself, rather than from ischemia alone. This aligns with recent studies showing that thrombosis‐associated exosomes can transport regulatory RNAs capable of reshaping the metabolic and immunological state of distant cells [7–9], highlighting the importance of exosome‐mediated signalling in acute cardiovascular injury ([20]).

First, multiomics and epidemiological analyses indicate an elevated risk of T1MI in patients with lung cancer, with significant associations observed between these two diseases. This finding is consistent with recent multicentre epidemiological studies and further supports a shared genetic background for cancer and cardiovascular disease, suggesting that patients may be predisposed to both conditions through overlapping susceptibility pathways [4–6, 23]. Nevertheless, the specific molecular mechanisms by which cancer promotes cardiovascular events, especially the cross‐tissue and cross‐signalling axis crosstalk, remain to be further elucidated. Our data add an additional layer of evidence to the link between cancer and cardiovascular disease by highlighting FENDRR as a candidate lncRNA that may connect these two conditions at the molecular level. The bidirectional association between malignancy and cardiovascular risk has been recognised for many years, but only a small number of molecules have been shown to act in both settings with clearly demonstrable functional effects [24, 25]. In our study, the increase of exosomal FENDRR in T1MI and its association with immune‐related features in LUAD point to a shared, RNA‐mediated mode of communication, suggesting that systemic lncRNA signalling may contribute to comorbidity more than previously thought.

At the molecular level, high FENDRR expression in TEs and T1MI models was shown to drive iron‐dependent cell death and oxidative stress in cardiomyocytes, thereby promoting apoptosis and functional impairment through the regulation of core ferroptosis and ferritinophagy molecules such as NCOA4, GPX4 and P62. These results are in line with recent studies reporting the proinjury effects of lncRNA‐mediated ferroptosis in the context of myocardial damage [26–28]. Our animal experiments further showed that shFENDRR treatment partly reduced myocardial fibrosis and functional damage and improved the dysregulated expression of ferroptosis‐ and ferritinophagy‐related molecules induced by T1MI, indicating that FENDRR actively participates in the development of cardiac injury.

In contrast, comprehensive pan‐cancer omics and machine learning analyses indicated that FENDRR is generally downregulated in a wide range of solid tumours, and that higher expression is linked to better survival outcomes, especially in LUAD. This observation suggests that FENDRR may play a tumour‐suppressive role in cancer, possibly by modulating ferroptosis and immune responses. However, the underlying molecular mechanisms, including the regulation of iron metabolism and immune cell infiltration, need further validation. At first glance, this pattern contrasts with the role of FENDRR in T1MI, where it appears to worsen myocardial injury, and it implies that the actions of FENDRR vary across tissues and depend heavily on the local cellular and tissue environment [29]. The opposing patterns observed in T1MI and lung cancer indicate that FENDRR may exert different effects depending on the tissue context, contributing to myocardial injury in acute ischemic settings while reflecting a more favourable immune landscape in LUAD. However, the exact molecular pathways underlying these dual roles, including PRC2 binding and iron metabolism, remain to be fully elucidated. In cardiomyocytes, FENDRR promotes ferroptosis through the NCOA4–GPX4–P62 axis, thereby amplifying oxidative injury and functional decline [4–6]. This is consistent with our previous findings [4–6], in which FENDRR was shown to aggravate oxidative stress and disrupt iron‐handling pathways. In addition to our own data, several recent studies have also provided evidence that FENDRR plays an important role in regulating oxidative stress responses and metabolic homeostasis in cardiac cells, further supporting its involvement in myocardial injury ([30, 31]). In contrast, in tumours, ferroptosis can act as a tumour‐suppressive mechanism [32]; higher FENDRR expression may enhance this process and remodel the immune microenvironment in favour of anti‐tumour responses [27, 28]. A similar pattern has also been described for other lncRNAs, including MALAT1 and HOTAIR [24, 33], whose biological effects can even move in opposite directions when the tissue type or signalling environment changes. From a clinical perspective, these findings suggest that FENDRR may serve as a detrimental factor in cardiovascular settings but a protective biomarker in oncology, underscoring the need to integrate tissue context when considering lncRNAs as diagnostic or therapeutic targets. Such apparent contradictions may arise from FENDRR′s distinct positions within different signalling pathways, epigenetic landscapes, and protein–protein interaction networks.

Analysis of the immune microenvironment further demonstrated that high FENDRR expression is closely associated with increased expression of immune molecules such as CD8^+^ T cells, Treg and PTX3, suggesting its ability to promote T cell activation and immune inflammatory responses, while also modulating the distribution of immunosuppressive cell subsets [34]. The simultaneous presence of immune activation and immune suppression provides an important biological basis for the interaction between cancer and myocardial infarction and also offers a theoretical foundation for the development of immunotherapies and interventions targeting cardiovascular complications [35]. Our immunohistochemistry results further confirmed that, in the context of cancer, myocardial infarction thrombi exhibited significantly enhanced local infiltration of CD8^+^ T cells and Treg, as well as upregulation of PTX3, whereas CXCL8 and PD‐L1 levels remained largely unchanged, highlighting the complexity and diversity of the cancer–myocardial infarction microenvironment.

Moreover, single‐cell omics analyses revealed spatially resolved expression of FENDRR within stromal and certain immune cell subsets, supporting its role as a molecular bridge in cancer–myocardial infarction comorbidity via the immunity–ferroptosis–exosome axis. These mechanisms merit further investigation through spatial omics and advanced functional experiments in future studies.

Taken together, our data suggest that FENDRR does not have a single, fixed role across diseases. In the setting of T1MI, higher FENDRR expression in TEs is associated with increased oxidative stress, iron overload, cardiomyocyte ferroptosis and more severe myocardial injury. In lung cancer, by contrast, FENDRR expression is reduced, and in LUAD cohorts, patients with relatively higher FENDRR expression show better survival and a more active anti‐tumour immune microenvironment. These opposite patterns indicate that the biological effects of FENDRR are strongly shaped by the local tissue and microenvironment, and that the same lncRNA may worsen injury in the heart while supporting immune control of tumour growth in the lung. Understanding how FENDRR is integrated into these organ‐specific signalling networks will be important both for interpreting cancer–cardiovascular comorbidity and for judging whether FENDRR can be used as a biomarker or therapeutic target. Future studies combining larger clinical cohorts with in‐depth mechanistic and spatial analyses will be needed to clarify these questions and to define under which conditions modulation of FENDRR could be clinically beneficial.

## 5. Limitations

This study has several limitations that need to be considered. First, we integrated multiomics data and experimental models to infer potential mechanisms, the clinical sample size remains relatively small, limiting the generalizability and robustness of the findings. The current sample size may not fully capture the diversity of clinical presentations; therefore, future studies involving larger, multicentre cohorts will be required to substantiate these findings. Second, the reliance on omics‐based approaches carries inherent limitations, such as the potential bias introduced by data normalization and the complexity of integrating large‐scale datasets. Although the data suggest that FENDRR may influence ferroptosis and immune regulation, further experimental validation using biochemical assays and in vivo models is essential to confirm these inferences at a molecular level. Third, the physiological role of exosomal lncRNAs like FENDRR, particularly regarding their transport and functional significance in the circulation, requires more detailed exploration. Current understanding remains limited, and future research should focus on in situ models and expanded clinical datasets to provide a clearer understanding of their therapeutic potential. Lastly, although single‐cell RNA sequencing and omics data have provided insights into the potential roles of FENDRR in immune modulation, these findings should be corroborated by more refined single‐cell techniques and spatial omics analyses to define the cellular context in which FENDRR exerts its effects.

## 6. Conclusion

In summary, this study provides preliminary evidence for the genetic and molecular associations between lung cancer and T1MI, identifying elevated expression of FENDRR in TEs in T1MI, where it may promote ferroptosis and immune remodelling. In contrast, reduced FENDRR expression in lung cancer is associated with a more favourable prognosis, suggesting context‐ and tissue‐specific roles. These findings offer initial insights into the molecular mechanisms of cancer–cardiovascular comorbidity and point to FENDRR as a potential target for future diagnostic and therapeutic exploration, although validation in larger and independent cohorts will be essential.

## Ethics Statement

Ethical approval has been obtained for the collection and use of thrombi and blood from relevant patients for this study, which primarily involves human subjects. The ethical review of this study was obtained from the Ethics Committee of Guizhou Provincial People′s Hospital (Approval No. EC Review (Scientific Research) 2022‐79 (research involving humans) and Approval No. EC Review (animal) 2022‐017). SD rats ranging from 12 to 16 weeks in age were used for animal studies and were provided by Liaoning Changsheng Biotechnology Co. LTD. Use of animals was approved by the Ethics Committee of Guizhou Provincial People′s Hospital and conformed to the ‘Guide for the Care and Use of Laboratory Animals’ published by the US National Institutes of Health (NIH Publication No. 85‐23, revised 1996). The biological samples obtained for this study were obtained from patients at the Guizhou Provincial People′s Hospital. All patients had their written consent signed by themselves.

## Disclosure

All authors reviewed and approved the final version of the manuscript. The funders had no role in study design, data collection and analysis, decision to publish, or preparation of the manuscript.

## Conflicts of Interest

The authors declare no conflicts of interest.

## Author Contributions

Youfu He and Qiang Wu conceived and supervised the project. Youfu He and Yu Qian performed the majority of experiments and data analysis. Yu Zhou and Zhiwei Zheng contributed to bioinformatics analyses and manuscript preparation. Chen Li assisted with clinical data collection and animal studies. Youfu He drafted the initial manuscript, and Qiang Wu critically revised the paper.

## Funding

This work was supported by the Guizhou Provincial Science and Technology Agency Project (Qian Ke He Foundation ZK [2023] General 217; Qian Ke He Foundation ZK [2023] General 216); Key Advantageous Discipline Construction Project of Guizhou Provincial Health Commission in 2023; and Provincial Key Medical Discipline Construction Project of the Health Commission of Guizhou Province from 2025 to 2026.

## General Statement

*Cell Line Statement.* The AC16 cell line used in this study was purchased from Wuhan Pricella Biotechnology Co. Ltd. (Catalogue No. CL‐0790) in January 2024. The cell line was authenticated by the supplier at the time of shipment and confirmed to be free of mycoplasma contamination. (RRID:CVCL_4U18).

## Supporting Information

Additional supporting information can be found online in the Supporting Information section.

## Supporting information


**Supporting Information 1** Figure S1. Additional immune activity analyses associated with FENDRR expression. (a) Comparison of chemokine‐related signatures between high‐ and low‐FENDRR groups. (b) Cytolytic activity (CYT) scores stratified by FENDRR expression. (c) Differences in IFN*γ*‐associated signatures between FENDRR expression groups.

**Supporting Information 2** Figure S2. Differential expression and survival analysis of FENDRR‐related genes based on TCGA data. (a) Volcano plot of differential expression in LUAD with high versus low FENDRR. (b) Volcano plot of FENDRR‐related gene differential expression between LUAD and controls. (c) Forest plot of COX regression for FENDRR‐related genes in LUAD.

## Data Availability

The data that support the findings of this study are available from the corresponding author upon reasonable request.
